# Mycobacterium tuberculosis PPE51 Inhibits Autophagy by Suppressing Toll-Like Receptor 2-Dependent Signaling

**DOI:** 10.1128/mbio.02974-21

**Published:** 2022-04-25

**Authors:** Emily J. Strong, Jia Wang, Tony W. Ng, Steven A. Porcelli, Sunhee Lee

**Affiliations:** a Department of Microbiology and Immunology, University of Texas Medical Branch, Galveston, Texas, USA; b Department of Microbiology and Immunology, Albert Einstein College of Medicine, Bronx, New York, USA; c Department of Medicine, Albert Einstein College of Medicine, Bronx, New York, USA; d Department of Molecular Genetics and Microbiology, Duke University, Durham, North Carolina, USA; University of Massachusetts Amherst

**Keywords:** *Mycobacterium tuberculosis*, autophagy, host-microbe interaction, innate immunity, mitogen-activated protein kinase ERK1/2, Toll-like receptor 2

## Abstract

Autophagy is an ubiquitous homeostatic pathway in mammalian cells and plays a significant role in host immunity. Substantial evidence indicates that the ability of Mycobacterium tuberculosis (Mtb) to successfully evade immune responses is partially due to inhibition of autophagic pathways. Our previous screening of Mtb transposon mutants identified the PPE51 protein as an important autophagy-inhibiting effector. We found that expression of PPE51, either by infecting bacteria or by direct expression in host cells, suppressed responses to potent autophagy-inducing stimuli and interfered with bacterial phagocytosis. This phenotype was associated with reduced activation of extracellular signal-regulated kinase 1/2 (ERK1/2), a key component of signaling pathways that stimulate autophagy. Multiple lines of evidence demonstrated that the effects of PPE51 are attributable to signal blocking by Toll-like receptor 2 (TLR2), a receptor with known involvement of activation of ERK1/2 and autophagy. Consistent with these results, mice with intact TLR2 signaling showed striking virulence attenuation for an Mtb *ppe51* deletion mutant (Δ*51*) compared to wild-type Mtb, whereas infection of TLR2-deficient mice showed no such attenuation. Mice infected with Δ*51* also displayed increased T cell responses to Mtb antigens and increased autophagy in infected lung tissues. Together, these results suggest that TLR2 activates relevant host immune functions during mycobacterial infection, which Mtb then evades through suppression of TLR2 signaling by PPE51. In addition to its previously identified function transporting substrates across the bacterial cell wall, our results demonstrate a direct role of PPE51 for evasion of both innate and adaptive immunity to Mtb.

## INTRODUCTION

Mycobacterium tuberculosis (Mtb) is one of the most successful host-adapted pathogens globally. In 2020 alone, 1.5 million people, including 214,000 HIV-positive individuals, died from Mtb infection, while a further 10 million people developed tuberculosis (TB) diseases (1). Although there are effective drugs against Mtb, treatment timelines are 6 months or longer, and the rise of multidrug-resistant (MDR) and extensively drug-resistant (XDR) infections demonstrates the urgent need for new and improved treatment options (1). A better understanding of host pathways affected by Mtb will potentially lead to better opportunities for developing successful host-directed therapies for the treatment and prevention of TB (2–4). Some of the host-directed therapies currently under consideration for use against Mtb involve activating the autophagy pathway (2), a fundamental cellular process that pathogens must overcome upon invasion of eukaryotic cells. Ubiquitin targeting of intracellular bacteria, including Mtb, plays a fundamental role in a selective form of autophagy called xenophagy, constituting a crucial innate immune mechanism in mammalian cells. Induction of autophagy by pharmacological means during Mtb infection enhances bactericidal effects. Autophagy can initiate phagosome maturation and the presentation of processed antigens to T cells (5–7). The activation and regulation of autophagy are complex and affected by many feedback mechanisms, not all of which are fully understood, especially in the context of mycobacterial infection (8–10).

The members of the PE/PPE family of mycobacterial proteins are found most abundantly in slow-growing pathogenic mycobacteria and constitute approximately 10% of the coding capacity of the Mtb genome (11). It has been previously demonstrated that the Mtb PE/PPE proteins are partially responsible for inhibiting autophagy in phagocytic cells upon infection with mycobacteria (12–15). This family of proteins coevolved with the ESX type VII secretion systems in mycobacteria, which are prominently involved in mycobacteria's virulence and intracellular survival (16, 17). Several PE/PPE family members have been implicated as inhibitors of autophagy in Mtb-infected cells, thus contributing to the virulence and persistence of the bacteria. Among these, our previous work identified PPE51, which has been extensively evaluated as a critical component of membrane and cell wall transport of carbon substrates and a mediator of pH-dependent growth of Mtb (18–20), as a potentially important autophagy inhibiting factor (12).

Other mycobacterial proteins have also been identified in modulating the autophagy pathway (4, 13, 15, 21–25). Mtb cytosolic DNA is recognized by the cytosolic DNA sensor, cyclic GMP-AMP synthase, leading to type I interferon release and recruitment of autophagy receptors such as p62 (26, 27). EspB is part of the ESX1 secretory apparatus and have been shown to downregulate the gamma interferon (IFN-γ) receptor, resulting in STAT activation (28). Similarly, the enhanced intracellular survival (*eis*) gene of Mtb inhibits autophagy by inhibiting the activation of Jun N-terminal kinase (JNK) and reactive oxygen species (ROS) generation (23). Alternatively to canonical autophagy inhibition, PknG induces canonical autophagy but inhibits autophagy flux by targeting Rab14 (29). Our recent studies also demonstrated that PE_PGRS20 and PE_PGRS47 inhibit autophagy in macrophages via association with Rab1A (30).

In the current study, we focused on the function of PPE51 as a significant inhibitor of autophagy during infection and provide evidence linking this function to signaling through the innate immunity receptor, Toll-like receptor 2 (TLR2). Autophagy induction and bacterial survival in phagocytic cells were examined for an Mtb *ppe51* deletion mutant (Δ*51*). We also assessed the effects of PPE51 on infection in mice, which confirmed the contribution of PPE51 to virulence and inhibition of autophagy and priming adaptive immune responses *in vivo*. These results establish an essential role for PPE51 in promoting virulence and immune evasion by Mtb through effects on TLR2 signaling, resulting in autophagy inhibition and reduction of innate and adaptive immunity.

## RESULTS

### Inhibition of autophagy and enhancement of mycobacterial survival by PPE51.

Recently, PPE51 was shown to have an essential role in maintaining the survival of Mtb in the presence of simple carbon sources and acidic pH (18–20), indicating that PPE51 plays a pivotal role in the bacterium's response to starvation and stress that is likely to be relevant to conditions encountered during host infection. Through a loss-of-function screen of a transposon mutant library, we previously identified PPE51 as also functioning as an autophagy inhibitor (12). To confirm and further evaluate the role of PPE51 in host-pathogen interactions, we created a targeted *ppe51* deletion in Mtb H37Rv (Δ*51*) via homologous recombination using specialized transduction as previously described (31). We assayed the *in vitro* growth of this strain in complete medium (7H9) at pH 7.4 or pH 5.4 or in minimal medium (Sauton) at pH 5.4 or pH 7.4 with glycerol as the sole carbon source (Fig. S1A to D). Consistent with previous observations, deletion of *ppe51* did not alter bacterial growth in complete medium at either neutral or acidic pH by Δ*51* compared to wild-type (WT) Mtb. However, in minimal medium with glycerol as the only carbon source, Δ*51* demonstrated enhanced growth at pH 5.4 but markedly diminished growth at neutral pH compared to WT bacteria. These growth effects were reversed by genetic complementation (Δ*51*C). Similarly, an unbiased forward genetic screen previously discovered that the PPE51 is required to arrest Mtb growth at acidic pH on specific carbon sources (20).

10.1128/mBio.02974-21.2 FIG S1 Mtb PPE51 is required for growth in the presence of glycerol as a sole carbon source at neutral pH but not acidic pH. Growth curves of WT, Δ*51,* and Δ*51*C were determined in 7H9 (A), 7H9 pH 5.4 (B), minimal medium at pH 5.4 (C), and minimal medium at pH 7.4 with 0.2% glycerol as the sole carbon source (D). Data are mean absorbances and SD of duplicate cultures from one of three representative experiments. Significance was calculated by two-way ANOVA corrected by Dunnett's test for multiple comparisons. (E) One-dimensional thin-layer-chromatographic analysis of petroleum ether soluble lipids of Mtb, Δ*ppe51*, and complement strains is shown. *, *P* < 0.05; **, *P* < 0.01; ***, *P* < 0.001. Download FIG S1, TIF file, 2.2 MB. Copyright © 2022 Strong et al. 2022 Strong et al. https://creativecommons.org/licenses/by/4.0/ This content is distributed under the terms of the Creative Commons Attribution 4.0 International license.

It has also been observed that defects in growth and uptake of carbon substrates by *ppe51*-deficient strains can be reversed by spontaneous mutations in the pathway for synthesis or export of the cell wall glycolipid phthiocerol dimycocerosate (PDIM) (19). To confirm that our *ppe51* mutant had not lost the ability to synthesize PDIM, we examined the apolar lipids of the bacteria by thin-layer chromatography (TLC). No apparent differences were observed by TLC in the apolar lipid PDIM-containing fraction of WT Mtb, Δ*51*, or Δ*51*C strains (Fig. S1E). This result was further supported by the reduced growth of Δ*51* at neutral pH in a simple carbon source, since Wang et al. demonstrated that defective PDIM synthesis allows normal growth of *ppe51* mutants under these conditions (19).

**FIG 1:**
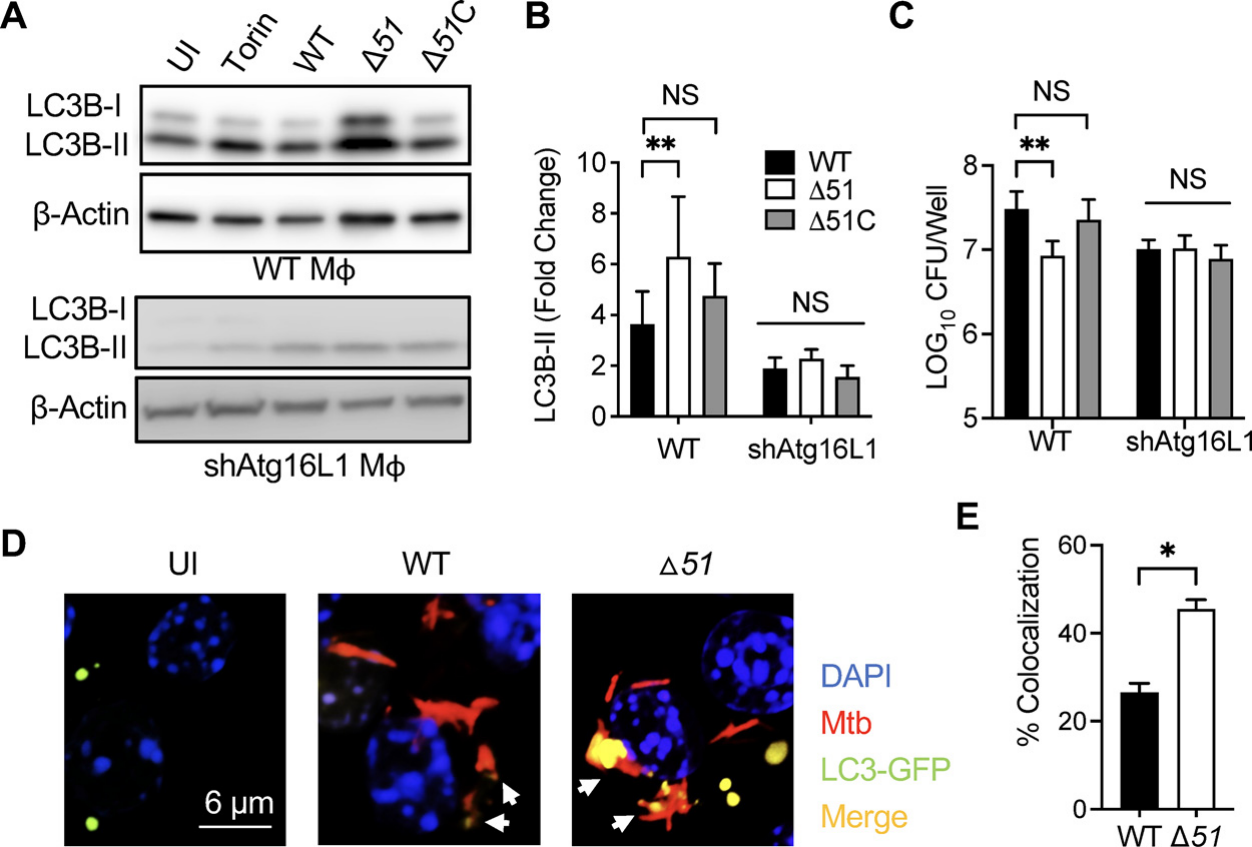
Mtb PPE51 inhibits canonical autophagy, resulting in increased bacterial survival. (A) Immunoblot of LC3B accumulation in RAW 264.7 macrophages and RAW 264.7 shAtg16L1 macrophages 24 h after infection with WT, Δ*51*, and Δ*51*C at an MOI of 10 and macrophages that were uninfected (UI) or Torin (10 μM) treated. A representative blot is shown for three independent assays. (B) The densitometric summary analysis was calculated by LC3B-II density normalized to β-actin density, and then the fold change ratio was calculated compared to the uninfected control for each assay. (C) The survival of mycobacteria was determined in WT and shAtg16L1-deficient macrophages. (D) Confocal microscopy images were taken 24 h after infection of RAW 264.7 LC3B-GFP macrophages with Mtb::DsRed and Δ*51*::DsRed at an MOI of 10 and macrophages that were uninfected (UI). (E) Colocalization of LC3B-GFP puncta and DsRed bacteria was quantified, and Pearson’s correlation coefficient was calculated. Data are representative of three independent experiments, and significance was calculated by two-way ANOVA corrected by Dunnett's test for multiple comparisons or unpaired *t* test. *, *P* < 0.05; **, *P* < 0.01; NS, not significant.

To confirm our previous findings using a transposon mutant with a mutation in the *ppe51* gene (30), we used Δ*51* to assay autophagy and bacterial survival in macrophages. Autophagy was increased in the RAW 264.7 macrophage cell line infected with Δ*51* compared to WT or Δ*51*C infection (Fig. 1A and B). To confirm that the increased autophagy with *Δ51* was reliant upon canonical autophagy, we analyzed the impact of genetic knockdown of the autophagy-related gene (ATG) 16L1, an essential component of this pathway. Infection of RAW 264.7 cells transfected with a small hairpin inhibitory RNA targeting ATG16L1 (shAtg16L1 macrophages [Mϕ]) demonstrated limited autophagy even during Δ*51* infection (Fig. 1A and B). Autophagy has been established to be an efficient way of clearing intracellular mycobacteria (6, 7, 12, 13), and consistent with this, we observed decreased intracellular bacterial burden 24 h postinfection in RAW 264.7 macrophages with Δ*51* infection than infections with WT or Δ*51C* strains (Fig. 1C). In contrast, Atg16L1 silencing resulted in similar intracellular numbers following infections with all bacterial strains. We further investigated the autophagosome formation by confocal microscopy (Fig. 1D and E) and flow cytometry (Fig. S2), confirming the enhanced autophagy induction during Δ*51* infection of macrophages.

10.1128/mBio.02974-21.3 FIG S2 Mtb PPE51 decreases the mean fluorescence intensity (MFI) of LC3B-GFP. Histograms indicating the MFI of RAW 264.6 macrophages expressing LC3-GFP infected with WT, Δ*51,* and Δ*51*C are shown. MFI data were obtained by 488-nm excitation and 530/30-nm emission using the BD Accuri C6 flow cytometer at 24 h postinfection. Means and SD of a representative of 3 independent experiments are shown. Significance was calculated by one-way ANOVA corrected by Dunnett's test for multiple comparisons. ***, *P* < 0.001; ns, not significant. Download FIG S2, TIF file, 0.6 MB. Copyright © 2022 Strong et al. 2022 Strong et al. https://creativecommons.org/licenses/by/4.0/ This content is distributed under the terms of the Creative Commons Attribution 4.0 International license.

### Role of ROS generation and MAP kinases in autophagy induction by Δ*51*.

**FIG 2:**
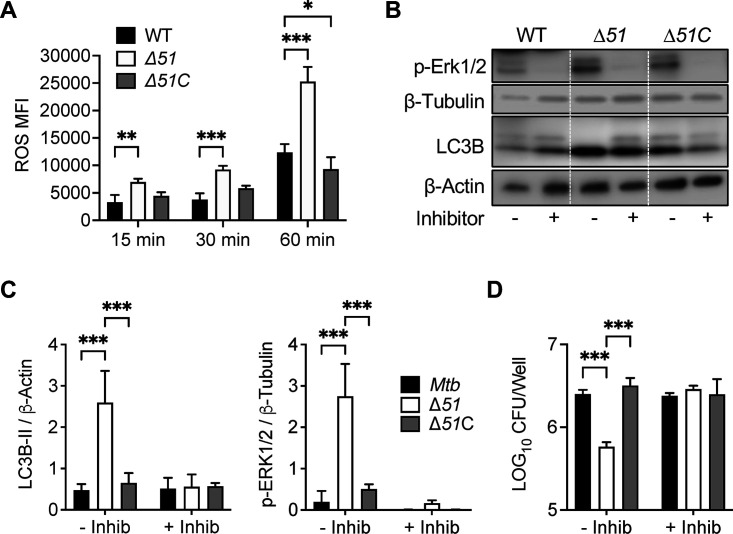
Role of ROS generation and MAP kinases in autophagy induction by Δ51. (A) RAW 264.7 macrophages treated with the oxidative stress reagent CellROX were infected with WT, Δ*51*, and Δ*51*C at an MOI of 10. At 15 min, 30 min, and 60 min, cells were collected and acquired by flow cytometry. (B) Immunoblot of phosphorylated ERK1/2 and LC3B accumulation in RAW 264.7 macrophages infected with WT, Δ*51*, and Δ*51*C at an MOI of 10, with or without ERK1/2 inhibitor (5 μM, FR180204) 24 h postinfection. A representative blot of three independent assays is shown. (C) The densitometric summary analysis was calculated by p-ERK1/2 density normalized to β-tubulin density and LC3B-II density to β-actin. (D) WT, Δ*51*, and Δ*51*C Mtb survival was determined in RAW 264.7 macrophages (MOI, 10) with or without ERK1/2 inhibitor (5 μM) at 24 h postinfection. All graphs represent one of three independent experiments, data are means and standard deviations (SD). Significance was calculated by two-way ANOVA corrected by Dunnett's test for multiple comparisons. *, *P* < 0.05; ***, *P* < 0.001.

ROS generated from both mitochondria and NADPH oxidases have been shown to activate autophagy to protect cells from nutrient starvation, dysfunctional mitochondria, cell death, and invading pathogens (32). Indeed, the Mtb enhanced intracellular survival protein (EIS) has been shown to inhibit ROS accumulation in macrophages resulting in autophagy inhibition (23). We examined ROS accumulation in RAW 264.7 macrophages by flow cytometry at 10, 30, and 60 min after infection with WT, Δ*51*, or Δ*51*C bacteria. Macrophages infected with Δ*51* exhibited significantly increased ROS levels at all three time points compared to those with WT and Δ*51*C infection (Fig. 2A). Since the MAP kinase Erk1/2 has been implicated as a downstream effector activated by ROS (33), we also assessed Erk1/2 phosphorylation in RAW 264.7 macrophages following infection with WT, Δ*51*, or Δ*51*C. This revealed a significant increase in Erk1/2 phosphorylation in macrophages infected with Δ*51* compared to WT and Δ*51*C. The addition of an Erk1/2-specific inhibitor prevented this increased phosphorylation and reduced autophagy, as assessed by LC3B accumulation (Fig. 2B and C), and also significantly increased the intracellular bacterial burden of Δ*51* at 24 h postinfection while not affecting the intracellular burden of WT or Δ*51*C strains (Fig. 2D). While some studies have identified the JNK mitogen-activated protein kinase (MAPK) or p38 MAPK as inhibiting ROS-induced stress in macrophages infected with mycobacteria (23, 34, 35), we found no differences in the expression or phosphorylation of these MAPKs during Δ*51* infection compared to WT and Δ*51*C (Fig. S3).

10.1128/mBio.02974-21.4 FIG S3 Mtb PPE51 does not affect phosphorylation of MAPKs p38 and SAPK/JNK. A representative immunoblot of phosphorylated p38 and SAPK/JNK accumulation in RAW 264.7 macrophages infected with WT, Δ*51,* and Δ*51C* at an MOI of 10, 24 h postinfection, is shown. A representative blot of three independent assays is shown. Download FIG S3, TIF file, 0.9 MB. Copyright © 2022 Strong et al. 2022 Strong et al. https://creativecommons.org/licenses/by/4.0/ This content is distributed under the terms of the Creative Commons Attribution 4.0 International license.

Given the effects of autophagy on regulating cellular homeostasis and cell death, the influence of PPE51 on apoptosis and necrosis during mycobacterial infection was examined in human macrophage-like THP-1 cells. We confirmed that Δ*51* induced more autophagy (Fig. S4A and B) and reduced intracellular bacterial burden compared to WT and Δ*51C* (Fig. S4C) in THP-1 macrophages, similar to our findings with RAW 264.7 macrophages. At 72 h postinfection, Δ*51* induced significantly more necrotic cell death than WT or Δ*51*C (Fig. S4D). This result was consistent with the increased ROS production with Δ*51* infection, given the known role of ROS as potent inducers of both autophagy and cell death (36).

10.1128/mBio.02974-21.5 FIG S4 Mtb PPE51 inhibits both autophagy and cell death in THP-1 cells. (A) Immunoblot of LC3B accumulation in THP-1 macrophages infected with WT, Δ*51*, and Δ*51*C at an MOI of 10 at 24 h postinfection is shown. A representative blot of three independent assays is shown. (B) The densitometric summary analysis was calculated by LC3B-II density normalized to β-actin density, and then the fold change ratio was calculated relative to the uninfected control for each assay. (C) WT, Δ*51*, and Δ*51*C survival was determined in THP-1 macrophages (MOI, 10) at 24, 48, and 72 h postinfection. (D) THP-1 cells were infected with WT, Δ*51*, and Δ*51*C at an MOI of 10 for 48 h. Cells were stained with annexin V-EnzoGold (PE) and necrosis detection reagent (peridinin chlorophyll protein [PerCP]). Cells were acquired by flow cytometry, and the percent PE-positive cells (apoptotic) or PerCP-positive cells (necrotic) were determined. All graphs represent one of three independent experiments, with data expressed as means and SD. Significance was calculated by one-way (B) or two-way ANOVA (C and D) corrected by Dunnett's test for multiple comparisons. *, *P* < 0.05; **, *P* < 0.01. Download FIG S4, TIF file, 0.4 MB. Copyright © 2022 Strong et al. 2022 Strong et al. https://creativecommons.org/licenses/by/4.0/ This content is distributed under the terms of the Creative Commons Attribution 4.0 International license.

### TLR2 dependence of inhibition of phagocytosis by PPE51.

**FIG 3:**
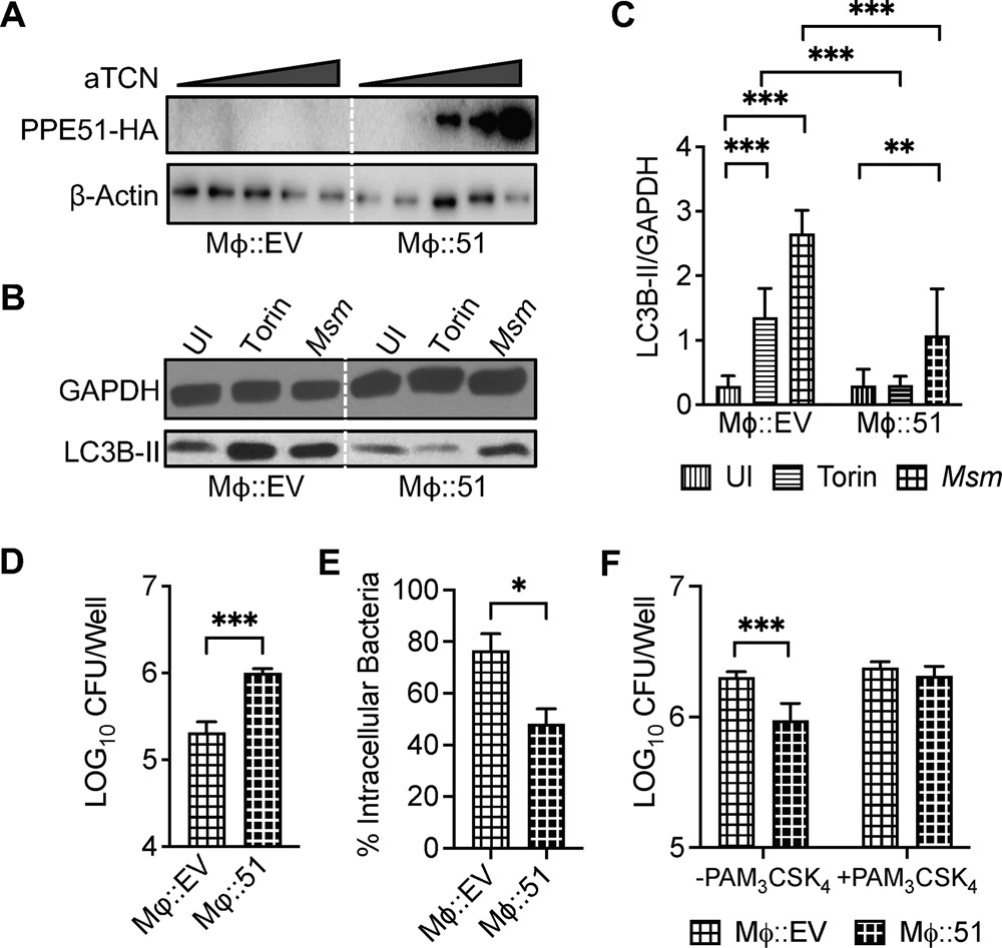
Macrophages expressing Mtb PPE51 inhibited autophagy and enhanced intracellular bacterial survival and phagocytosis. (A) THP-1 cells transduced with empty vector (Mϕ::EV) or HA-tagged PPE51 (Mϕ::51) were induced for 16 h with aTCN at 0, 125, 250, 500, or 1,000 ng/mL. Lysates were analyzed by Western blotting using an anti-HA antibody to detect PPE51 as a 40-kDa band. (B) Immunoblot of LC3B accumulation in Mϕ::EV and Mϕ::51 at 3 h after treatment with 10 μM Torin-1 or Msm (MOI 10) or uninfected (UI) macrophages. A representative blot of 3 independent assays is shown. (C) The densitometric summary analysis was calculated by LC3B-II density normalized to GAPDH density. (D) Msm survival was determined in Mϕ::EV and Mϕ::51 at 20 h postinfection. (E) Msm uptake by Mϕ::EV and Mϕ::51 (MOI 10) at 4 h postinfection was calculated by the ratio of intracellular bacteria to the inoculum. (F) Msm uptake by Mϕ::EV and Mϕ::51 (MOI, 10) with or without 100 ng/mL Pam_3_CSK_4_ at 4 h postinfection was determined. All graphs represent one of three independent experiments, with data expressed as means and SD. Significance was calculated by two-way ANOVA (corrected by Dunnett's test for multiple comparisons (C and F) or unpaired *t* test (D and E). *, *P* < 0.05; **, *P* < 0.01; ***, *P* < 0.001.

To assess the direct role of Mtb PPE51 in inhibiting autophagy as opposed to indirect effects related to changes in the bacteria as a result of PPE51 deletion, we generated a cell line (Mϕ::51) stably expressing PPE51 under the control of a tetracycline-inducible promoter. Upon induction with increasing anhydrous tetracycline concentrations, we observed expression of hemagglutinin (HA)-tagged PPE51 in lysates of the stably transfected cells by Western blotting (Fig. 3A). When treated with potent autophagy inducers, such as Torin-1 (37) or Mycobacterium smegmatis (Msm) (38), Mϕ::51 with induced expression of PPE51 demonstrated significantly less autophagy (Fig. 3B and C) and less clearance of intracellular Msm (Fig. 3D) than the empty-vector-transduced control macrophages (Mϕ::EV). To determine if PPE51 affected phagocytosis and thus survival, Mϕ::51 expressing PPE51 upon induction with anhydrous tetracycline (aTCN) was utilized to assess the direct role of Mtb PPE51 in phagocytosis. Mϕ::51 cells did not internalize Msm as efficiently as the control mock-transfected Mϕ::EV cell line (Fig. 3E), suggesting an inhibitory effect of PPE51 on phagocytosis. Similarly, the primary murine bone marrow-derived macrophages (BMDM) and dendritic cells (BMDDC) also demonstrated enhanced internalization of Δ*51* compared to WT or Δ*51*C (Fig. S5D and H). BMDM or BMDDC infected with Δ*51* underwent increased autophagy (Fig. S5A, B, E, and F) and reduced intracellular bacterial survival (Fig. S5C and G) during Δ*51* infection compared to WT or Δ*51*C infection. These results provided evidence that autophagy inhibition and enhanced intracellular bacterial survival were mediated directly by PPE51, independent of other host regulating effectors of Mtb.

10.1128/mBio.02974-21.6 FIG S5 Mtb PPE51 inhibits autophagy and phagocytosis, resulting in reduced bacterial burden in primary murine cells. (A) Immunoblot of LC3B accumulation in BMDM infected with WT, Δ*51,* and Δ*51*C at an MOI of 10, 24 h postinfection. A representative blot of 3 independent assays shown. (B) Densitometric summary analysis was calculated by LC3B-II density normalized to β-actin density, and then the fold change ratio was calculated relative to the uninfected control for each assay. (C) WT, Δ*51*, and Δ*51*C survival was determined in BMDM (MOI, 10) at 24 h postinfection. (D) WT, Δ*51*, and Δ*51*C (MOI, 10) uptake was determined at 4 h postinfection. The percentage of intracellular bacteria was determined by calculating the ratio of intracellular bacteria at 4 h postinfection to the inoculum. (E) Representative immunoblot of LC3B accumulation in BMDDC infected with WT, Δ*51*, and Δ*51*C at an MOI of 10, 24 h postinfection. A representative blot of 2 independent assays is shown. (F) Densitometric summary analysis was calculated by LC3B-II density normalized to β-actin density, and then the fold change ratio was calculated relative to the uninfected control for each assay. (G) WT, Δ*51*, and Δ*51*C survival was determined in BMDDC (MOI, 10) at 24 h postinfection. (H) WT, Δ*51*, and Δ*51*C (MOI, 10) uptake was determined at 4 h postinfection. The percent intracellular bacteria was determined by calculating the ratio of intracellular bacteria 4 h postinfection relative to the inoculum. All graphs represent one of three independent experiments, with data expressed as means and SD. Significance was calculated by one-way ANOVA corrected by Dunnett's test for multiple comparisons. *, *P* ≤ 0.05; **, *P* ≤ 0.01; ***, *P* ≤ 0.001. Download FIG S5, TIF file, 0.6 MB. Copyright © 2022 Strong et al. 2022 Strong et al. https://creativecommons.org/licenses/by/4.0/ This content is distributed under the terms of the Creative Commons Attribution 4.0 International license.

Several TLRs have been reported to stimulate autophagy in murine and human phagocytes (39), and signaling by agonist-stimulated receptors such as TLRs and FcγR during phagocytosis was shown to induce autophagosome formation and promote autophagosomal maturation (40). To assess if the effect of PPE51 on phagocytosis of mycobacteria was TLR dependent, immortalized bone marrow-derived macrophage cell lines derived from either wild-type mice or gene knockout mice lacking expression of TLR2, TLR4, or TLR9 were infected with Msm expressing empty vector (Msm::EV) or PPE51 (Msm::51) at multiplicities of infection (MOI) of 5 and 10 for 3 h. Deletion of TLR2 demonstrated a significantly decreased uptake of Msm::EV compared to WT macrophages, while deletion of TLR4 or TLR9 showed no significant effect. In contrast, a significant difference was not observed between Msm::EV and Msm::PPE51 uptake in TLR2-deficient macrophages but was found in the WT, TLR4^−/−^, and TLR9^−/−^ macrophages (Fig. S6A and B). To assess this further, we examined the effects of the TLR2 agonist Pam_3_CSK_4_ during Mϕ::51 infections with Msm. Pam_3_CSK_4_ treatment overcame PPE51-dependent suppression of bacterial uptake by the macrophages (Fig. 3F). These findings indicated a role for TLR2 in the phagocytosis of Mtb that was inhibited by PPE51.

10.1128/mBio.02974-21.7 FIG S6 Mtb PPE51 inhibits TLR2-dependent phagocytosis. Msm uptake by WT, TLR2^−/−^, TLR4^−/−^, and TRL9^−/−^ macrophages at MOI of 5 (A) and 10 (B) was determined. The percent intracellular bacteria was determined by calculating the ratio of intracellular bacteria 3 h postinfection to the inoculum. All graphs represent one of two independent experiments, with data expressed as means and SD. Significance was calculated by two-way ANOVA corrected by Bonferroni’s test for multiple comparisons. *, *P* < 0.05; **, *P* < 0.01; ***, *P* < 0.001. Download FIG S6, TIF file, 0.2 MB. Copyright © 2022 Strong et al. 2022 Strong et al. https://creativecommons.org/licenses/by/4.0/ This content is distributed under the terms of the Creative Commons Attribution 4.0 International license.

**FIG 4:**
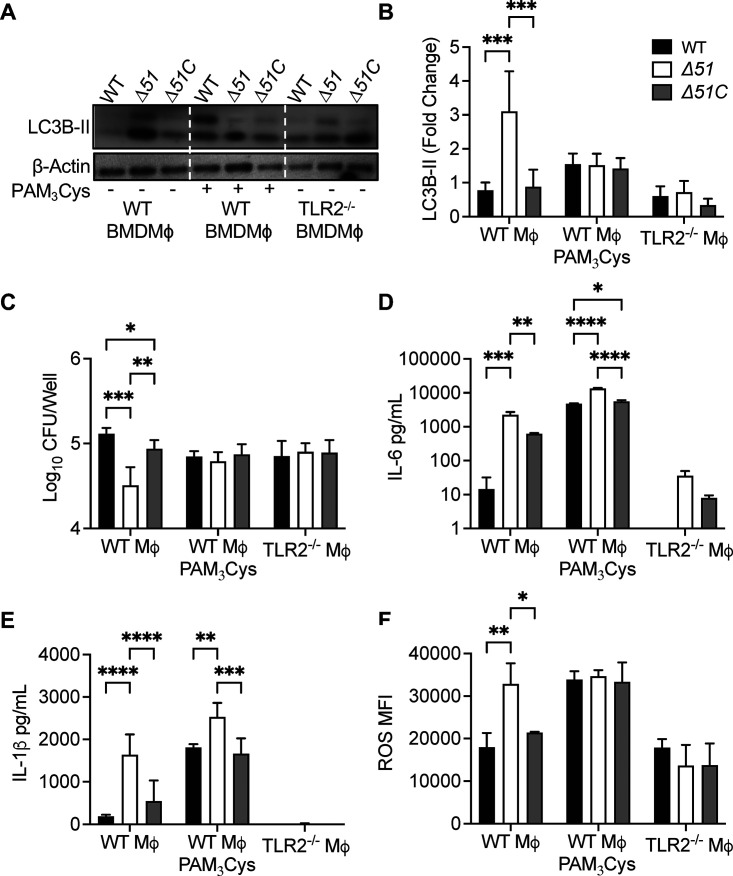
Mtb PPE51 inhibits TLR2 signaling. (A) Immunoblot of LC3B accumulation in WT BMDM with or without 100 ng/mL Pam_3_CSK_4_ and TLR2^−/−^ BMDM infected with WT, Δ*51*, and Δ*51*C at an MOI of 10 at 24 h postinfection. Representative blot of three independent assays shown. (B) The densitometric summary analysis was calculated by LC3B-II density normalized to β-actin density, and then the fold change ratio was calculated relative to the uninfected control for each assay. (C) WT, Δ*51*, and Δ*51*C survival was determined in WT BMDM with or without 100 ng/mL Pam_3_CSK_4_ and TLR2^−/−^ BMDM infected (MOI 10) at 24 h postinfection. (D and E) Cytokine concentration in WT BMDM with or without 100 ng/mL Pam_3_CSK_4_ and TLR2^−/−^ BMDM culture supernatant 24 h postinfection. (F) ROS accumulation in WT BMDM with or without 100 ng/mL Pam_3_CSK_4_ and TLR2^−/−^ BMDM 24 h postinfection. Macrophages were treated with CellROX 24 h postinfection, and ROS accumulation was determined by flow cytometry. The means and SD of representatives from three (B and C) or two (D to F) independent assays are shown. Significance was calculated by two-way ANOVA corrected by Dunnett's test for multiple comparisons (B to F). *, *P* < 0.05; **, *P* < 0.01; ***, *P* < 0.001; ****, *P* < 0.0001.

To confirm the effect of PPE51 on TLR2 signaling, we used primary BMDM isolated from wild-type or *tlr2*^−/−^ mice (Fig. 4). These were infected with WT, Δ*51*, or Δ*51*C. As we observed with similar infections of cell lines, increased autophagy was detected 24 h after infection of wild-type BMDM with Δ*51* compared to infection with WT or Δ*51*C. Primary macrophages from *tlr2*^−/−^ mice showed much lower autophagy during Δ*51* infection, which was similar to the low level of autophagy with infection using Mtb WT or Δ*51*C strains (Fig. 4A and B). Treatment with Pam_3_CSK_4_ partially reversed the inhibition of autophagy attributable to PPE51 in wild-type BMDM. As predicted, autophagy induction by Δ*51* in these BMDM resulted in reduced intracellular bacteria compared to WT or Δ*51*C infection. This reduced intracellular bacterial survival during Δ*51* infection was not observed in *tlr2*^−/−^ BMDM (Fig. 4C). Also similar to our results with macrophage cell lines, Δ*51*-infected WT primary BMDM secreted increased interleukin 6 (IL-6) and IL-1β and accumulated ROS, unlike in WT or Δ*51*C infection. Pam_3_CSK_4_ treatment significantly increased cytokine and ROS secretion and accumulation during all infections in WT BMDM. Conversely, *tlr2*^−/−^ BMDM tended to give only weak responses to infection with regard to cytokine secretion or ROS accumulation, and these did not differ between three Mtb strains (Fig. 4D to F). Taken together, these data suggest that PPE51 mediates multiple effects on infected host cells by inhibiting TLR2 signaling, resulting in the inability of infected cells to upregulate multiple host cell responses to infection, including autophagy, ROS accumulation, cytokine secretion, and intracellular mycobacterial killing.

### Requirement for PPE51 for virulence and evasion of adaptive immunity during infection *in vivo*.

**FIG 5:**
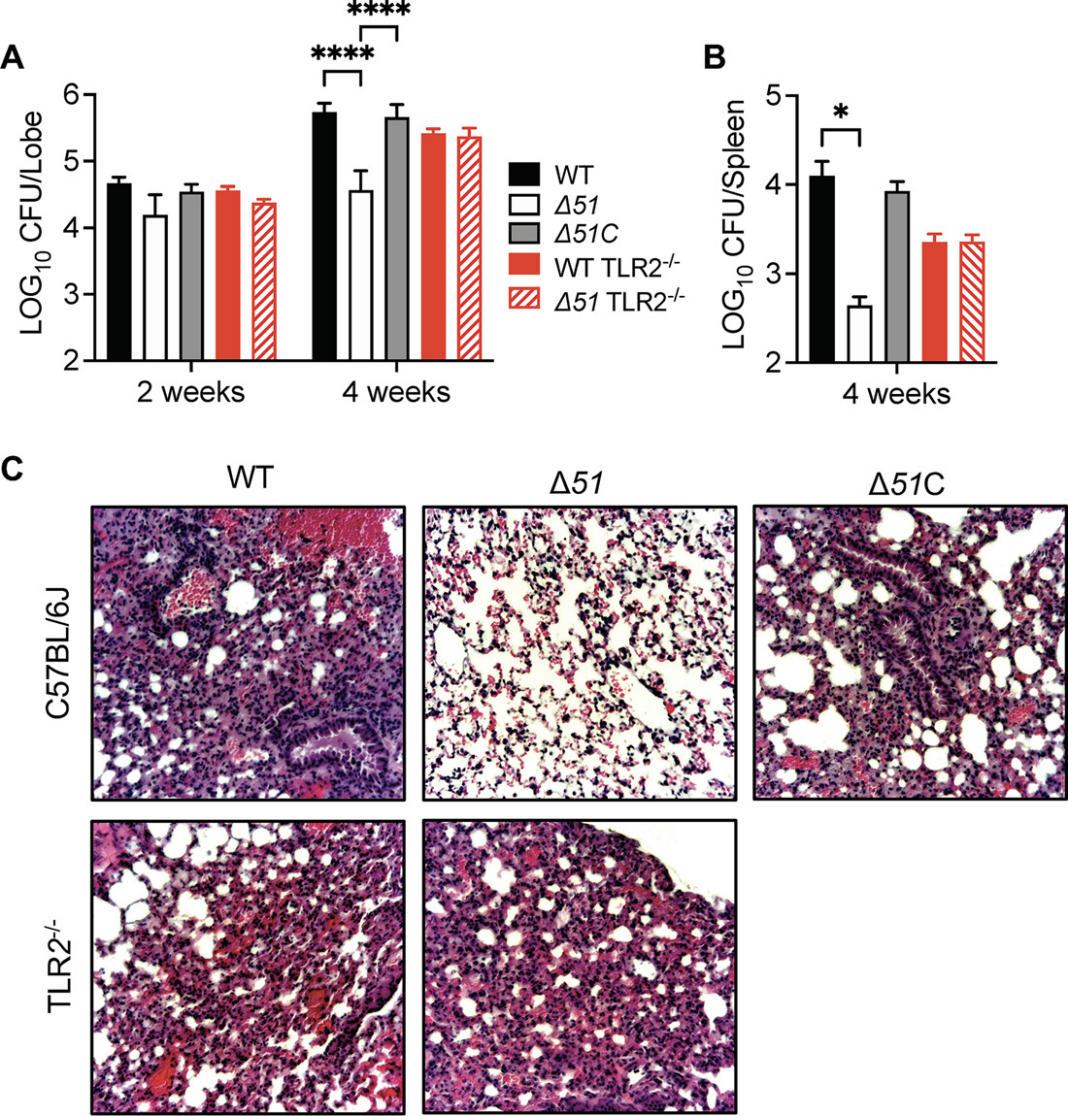
Mtb PPE51 is required for persistence *in vivo*. Mycobacterial load in the lungs (A) and spleens (B) of C57BL/6J or TLR2^−/−^mice infected via intranasal inoculation (∼200 CFU) with low doses of WT, Δ*51*, and Δ*51*C. Values are means and standard errors of the means (SEM) from two independent assays (*n* = 8 mice) and significance calculated by two-way ANOVA corrected by Dunnett's test for multiple comparisons. *, *P* < 0.05; ****, *P* < 0.0001. (B) Representative hematoxylin-and-eosin (H&E)-stained lung sections are shown for C57BL/6J or C57BL/6J TLR2^−/−^ mice at 4 weeks after infection with low dose intranasal of WT, Δ*51*, and Δ*51*C.

To examine the impact of PPE51 on Mtb survival and immunogenicity during infection *in vivo*, we infected wild-type or *tlr2*^−/−^ mice with ∼200 CFU of WT, Δ*51*, or Δ*51*C Mtb delivered to the lungs by intranasal inoculation. Determination of tissue bacterial counts at 2 or 4 weeks postinfection showed that Δ*51* was significantly attenuated in wild-type mice 4 weeks after infection (Fig. 5A and B). Histological examination of the lungs at 4 weeks postinfection showed reductions in leukocyte accumulation and the total area of histologically inflamed lung tissue in animals infected with the Δ*51* mutant compared to those with WT or Δ*51*C (Fig. 5C). In contrast, deletion of *ppe51* did not affect the growth of Mtb in harvested organs or lung pathology of infections in *tlr2*^−/−^ mice. These results were consistent with a significant role for PPE51 in the growth and survival of Mtb during acute infection *in vivo* by blocking the antibacterial effects resulting from TLR2 stimulation.

**FIG 6:**
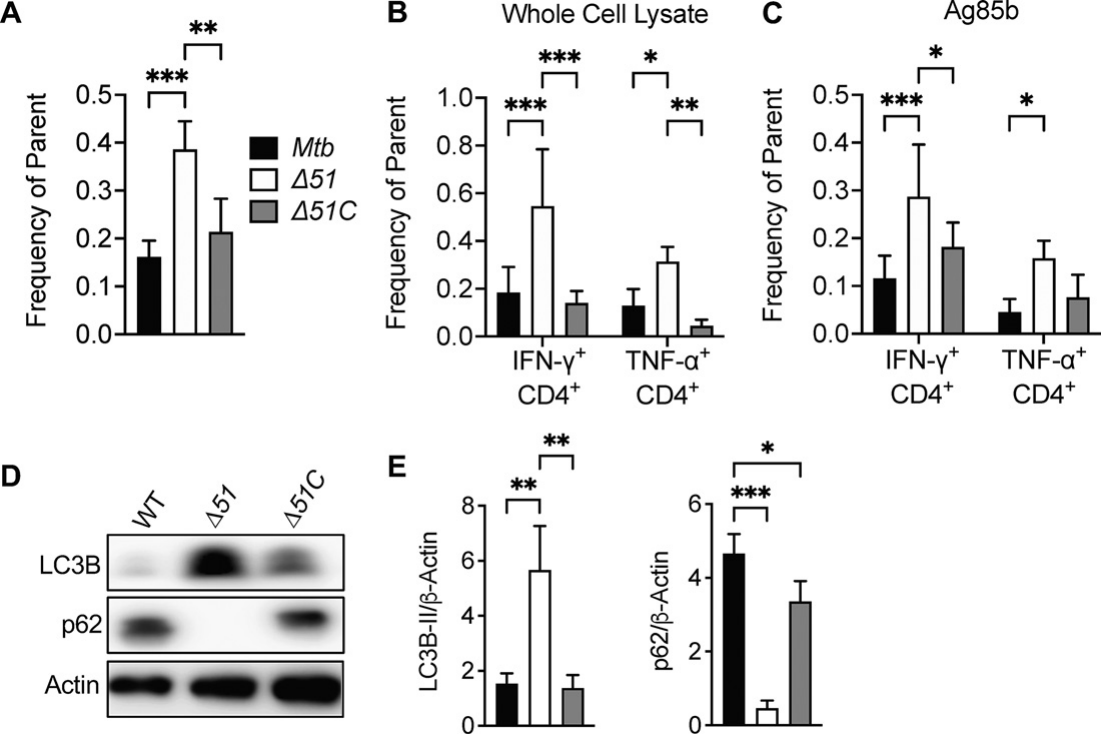
Mtb PPE51 is required for adaptive immune suppression in C57BL/6J mice. (A) Spleens from C57BL/6J mice infected with low intranasal doses (∼200 CFU) of WT, Δ*51*, and Δ*51*C were collected at 2 weeks postinfection. Cells were then stained with anti-CD4 MAb and MHC class II (I-A^b^) tetramers loaded with Ag85B peptide. The number of tetramer-positive CD4^+^ cells was determined by flow cytometry. (B and C) Splenocytes from infected mice were stimulated for 16 h with Mtb whole-cell lysate or Ag85b peptide. Stimulated cells were stained with anti-CD4 MAb and subsequently intracellularly stained for IFN-γ and TNF-α. The percentage of CD4^+^ cells staining for IFN-γ or TNF-α was determined by flow cytometry. (D) Right lower lung lobes from C57BL/6J mice were collected 4 weeks postinfection. Protein was extracted by TRIzol extraction and assayed by Western blotting for LC3B-II and p62 accumulation. A representative blot for one mouse per infection group from two independent experiments is shown (*n* = 8 mice). (E) The densitometric summary analysis was calculated by LC3B-II or p62 density normalized to β-actin density. Means and SD of representatives from two independent studies (A, B, C, and E) are shown. Significance was calculated by one-way ANOVA corrected by Dunnett's test for multiple comparisons. *, *P* < 0.05; **, *P* < 0.01; ***, *P* < 0.001.

Since autophagy has been identified as a route by which antigens can be processed and delivered to major histocompatibility complex (MHC) class II molecules, we assessed the ability of PPE51 to suppress MHC class II-dependent antigen presentation during Mtb infection *in vivo*. Two weeks following infection of C57BL/6 mice with WT, Δ*51*, or Δ*51*C, spleens from infected mice were collected and processed to obtain single-cell suspensions. The number of cells that bound MHC class II tetramers loaded with an immunodominant peptide of Ag85B were quantified using flow cytometry. We detected significantly more Ag85B-specific CD4^+^ T cells from spleens of mice infected with Δ*51* than from mice infected with WT or Δ*51*C (Fig. 6A). A similar finding was observed in suspensions from the lungs of wild-type mice (Fig. S7A). We also measured cytokine production by Mtb-specific CD4^+^ T cells by intracellular cytokine staining (ICS) to confirm increased responses in the Δ*51* infected mice. Splenocytes were stimulated with Mtb whole-cell lysate or Ag85B peptide and stained intracellularly for IFN-γ and tumor necrosis factor alpha (TNF-α). The number of IFN-γ- and TNF-α-producing CD4^+^ cells from Δ*51*-infected mice significantly increased in response to Mtb antigens compared with WT- and Δ*51*C-infected animals (Fig. 6B and C). These increased T cell responses appeared to be dependent on intact TLR2 signaling (Fig. S7B). Consistent with our *in vitro* studies, we observed significantly higher levels of LC3B-II accumulation and reduced p62 (SQSTM1) in the lungs of mice infected with Δ*51* compared to WT- or Δ*51*C-infected mice (Fig. 6D and E), confirming an increased induction of autophagy *in vivo* that may account for the enhanced CD4^+^ T cell responses through increased antigen processing and presentation.

10.1128/mBio.02974-21.8 FIG S7 Mtb PPE51 inhibits the *in vivo* adaptive immune response in a TLR2-dependent manner. (A) Lungs from WT C57BL/6J mice infected with low-dose intranasal (∼200 CFU) of WT, Δ*51*, and Δ*51*C were collected at 4 weeks postinfection. Then, cells were stained with anti-CD4 monoclonal antibody (MAb), and MHC class II (I-A^b^) tetramers loaded with Ag85B peptide. The number of tetramer-positive CD4^+^ T cells was determined by flow cytometry. Values are means and SD for four mice, and significance was calculated by one-way ANOVA corrected by Dunnett's test for multiple comparisons. *, *P* < 0.05. (B) Spleens from *tlr2*^−/−^ mice infected with low-dose (∼200 CFU) intranasal inoculation of WT, Δ*51*, or Δ*51*C were collected at 4 weeks postinfection. Splenocytes from infected mice were stimulated for 16 h with Mtb whole-cell lysate. Stimulated cells were stained with anti-CD4 MAb and subsequently intracellularly stained for IFN-γ and TNF-α. The percentages of CD4^+^ cells positive for IFN-γ or TNF-α staining were determined by flow cytometry. The values are means and SD for five mice, and significance was calculated by two-way ANOVA corrected by Dunnett's test for multiple comparisons. ns, not significant. Download FIG S7, TIF file, 0.1 MB. Copyright © 2022 Strong et al. 2022 Strong et al. https://creativecommons.org/licenses/by/4.0/ This content is distributed under the terms of the Creative Commons Attribution 4.0 International license.

## DISCUSSION

The current study provides data supporting the view that PPE51 is a bacterial effector that contributes to virulence of Mtb and evasion of host innate and adaptive immunity, most likely by acting mainly as an inhibitor of TLR2 signaling. PPE51 has been increasingly studied in the context of its roles in growth suppression at acidic pH and membrane permeability. Mutations in *ppe51* appear to impart an *in vitro* growth advantage in minimal medium at reduced pH and potentially act as selective channels, similar to outer membrane porins (18–20). We and others have reported that *ppe51* is upregulated at acidic pH in a *phoP*-dependent manner and downregulated under starvation conditions (12, 20). The restriction of growth at acidic pH in media with a defined carbon source (glycerol) is a trait associated with pathogenic bacteria, indicating that growth arrest during nutrient deprivation and low pH is essential for a full display of virulence (20, 41). Additionally, our current and previous studies have demonstrated that Mtb PPE51 plays an essential role in multiple virulence mechanisms used by Mtb to evade host immune responses and establish infection (12).

**FIG 7:**
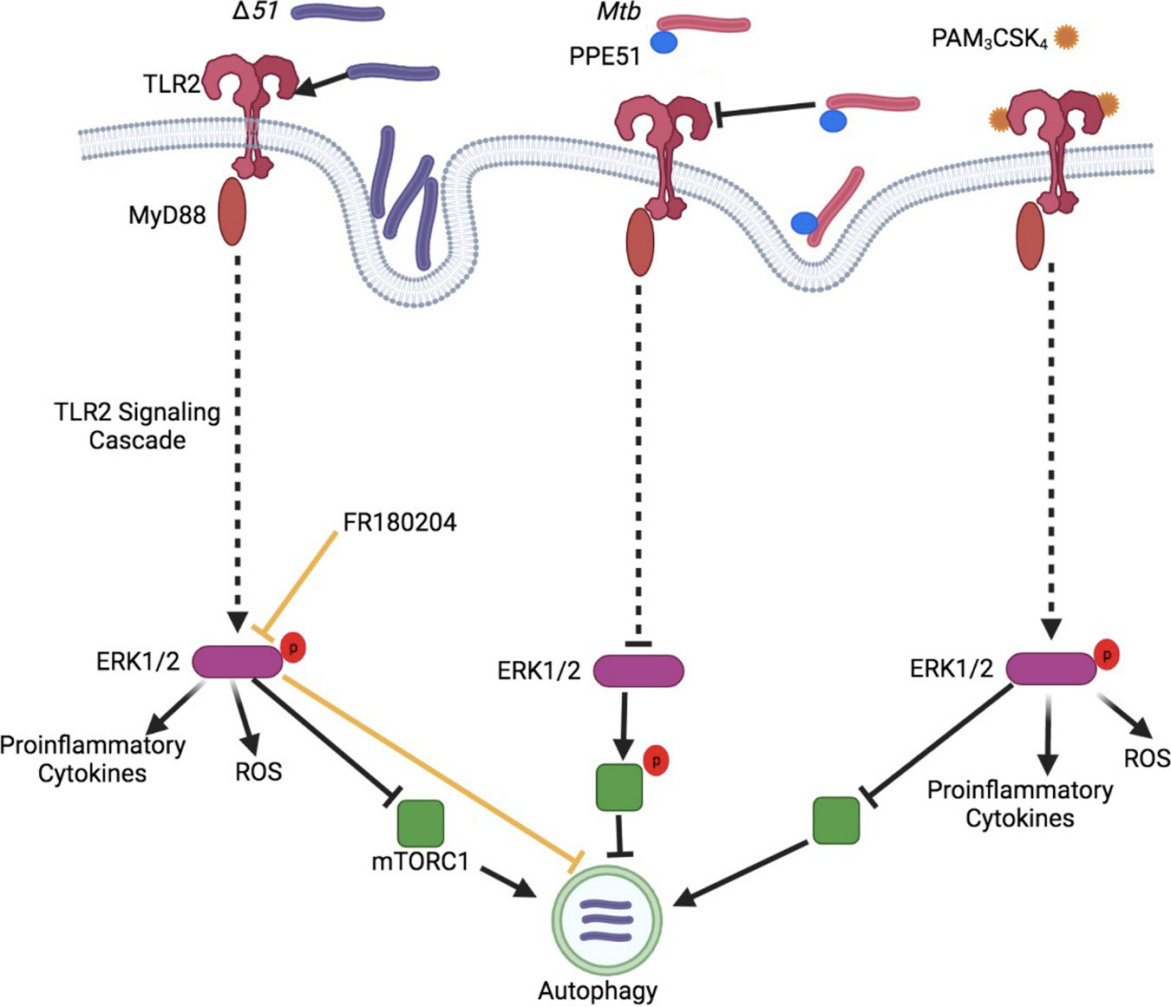
Proposed model of action for PPE51 inhibition of TLR2 signaling and autophagy. Data presented in the current study demonstrated that PPE51 inhibits TLR2-associated phagocytosis of Mtb and the associated signaling cascade. Infection of macrophages with Δ*51* demonstrated increased phosphorylation of MAP kinase, ERK1/2 resulting in enhanced proinflammatory cytokine secretion, ROS accumulation, and autophagy. Pam_3_CSK_4_ competitively overcame the PPE51 inhibition of this signaling cascade, resulting in bactericidal autophagy during Mtb infection. The image was generated using BioRender.com.

With recombinant Msm expressing Mtb PPE51 or the Mtb Δ*51* mutant, we reported that Mtb PPE51 is responsible for inhibiting autophagy and enhancing mycobacterial survival in infected macrophages (12). Here, we determined how PPE51 might suppress the autophagic pathway and lead to escape from autolysosomal degradation. Genetic deletion of *ppe51* from Mtb resulted in the enhanced activation of TLR2 signaling during infection, enhanced phosphorylation of MAPK Erk1/2 and accumulation of ROS and proinflammatory cytokines, increased autophagy, and reduced bacterial survival in macrophages. The accumulation of ROS likely led to increased cell death during Δ*51* infection. Based on our findings, we propose a model in which PPE51 interferes with TLR2 function, thus interfering with the TLR2 signaling cascade to reduce Erk1/2 MAPK activation, which generally would lead to increased autophagy, phagocytosis, MHC class II presentation, and cytokine production (Fig. 7). We believe that the inhibition of TLR2 signaling leading to ERK1/2 activation by PPE51 results in increased mTOR signaling and subsequent autophagy inhibition. In a previous study (12), we observed that infection with Msm expressing PPE51 results in increased mTOR phosphorylation and signaling aligning with decreased autophagy, further strengthening the hypothesis demonstrated in Fig. 7. As an alternative hypothesis, we investigated the possibility that PPE51 interacts with Atg8 (LC3)-family proteins. Atg8-interacting proteins contain a short linear LC3-interacting region/LC3 recognition sequence/Atg8-interacting motif (LIR/LRS/AIM) and are referred to as LIR-containing proteins (LIRCPs). To demonstrate if PPE51 is a LIRCP and interacts with ATG8, we have analyzed the PPE51 protein sequence with the iLIR database (https://ilir.warwick.ac.uk) for the xLIR motifs (42), but we did not find the motifs. Thus, we concluded that increased LC3 is not the result of the direct interaction of PPE51.

Many Mtb proteins, including some PE/PPE proteins, have been shown to induce apoptosis and autophagy via ROS and MAPK signaling in a TLR2-dependent manner (12, 13, 15, 23, 43–50). Unlike those observed here for PPE51, these interactions can result in augmented proinflammatory cytokine production and MHC class II antigen presentation (45–47). It was shown previously that PE_PGRS33 increases bacterial uptake of Mtb in a TLR2-dependent manner via the phosphatidylinositol 3-kinase pathway (48). Similarly, previous work demonstrated that PPE44 is involved in responses to multiple stresses, including oxidative and nutritional, and promotes macrophage expression of IL-12 p40 and IL-6 via the p38, ERK, and NF-κB signaling axis while increasing apoptosis of infected macrophages (12, 51). Unlike PPE51, there is no evidence that PPE44 is a TLR2 agonist, although we identified PPE44 as a possible inhibitor of autophagy in the same transposon mutant screen that identified this function for PPE51 (51). PPE26 has also been identified as a TLR2 agonist that stimulates cytokine production via activation of MAPK and NF-κB signaling (45).

Another TLR2 agonist, LpqT, signals through MAPK and inhibits MHC class II antigen presentation (46). Additionally, LPS, a potent bacterial effector, can induce TLR2 expression in murine macrophages (52), although TLR2 expression in human cells is not induced by such bacterial effectors (53). This lack of induction may be due to the reduced sensitivity of the TLR2 promoter in human cells to NF-κB activation, highlighting a potential avenue of further investigation for the role of PPE51 in the stimulation of NF-κB (53). While we propose that PPE51 exerts its functions mainly as a TLR2 antagonist, it could be proposed that a bacterial advantage exists if Mtb produces both agonistic and antagonistic TLR ligands, perhaps at different stages of infection or in different host microenvironments.

In contrast to the proinflammatory TLR signaling associated with many bacterial molecules, we observed TLR2 inhibition by PPE51, resulting in decreased bacterial uptake and reduced secretion of proinflammatory cytokines IL-6 and IL-1β (Fig. 4). We have observed that recombinant M. smegmatis expressing Mtb PPE51 (Msm::51) binds to the surface of macrophages in a TLR2-dependent manner, and the binding competes with synthetic TLR2 agonist Pam_3_CSK_4_ (data not shown). We speculate that PPE51 and Pam3CSK4 bind to TLR2 at the same regions of the lipopeptide binding pocket, which results in reduced Mtb phagocytosis. Future studies will directly evaluate a competition between purified PPE51 protein and Pam_3_CSK_4_. Autophagy inhibition by PPE51 was largely overcome in our *in vitro* experiments by activating TLR2 using the strong synthetic TLR2 agonist Pam_3_CSK_4_. As described above, differential regulation of TLR2 by murine and human macrophages may explain the variance in TLR2 activation by various Mtb PE/PPE proteins (43, 48, 53). Alternatively, these PE/PPE proteins may have independent roles in TLR2 signaling, either activating or inhibiting TLR2 signaling depending on the particular stage of infection or features of the tissue microenvironment.

The proinflammatory effects induced by TLRs are generally believed to support protective immune responses to mycobacterial infection (54–56). However, low-dose infection of Mtb in mice defective for TLR2 demonstrated little difference in inflammation, granuloma formation, or bacterial survival. As with studies demonstrating that silencing of essential autophagy machinery does not affect mycobacterial infection outcomes *in vivo* (57), these studies do not account for the ability of Mtb to inhibit TLR2 activation by PPE51 during infection. Whereas infection of WT mice with Δ*51* showed reduced tissue bacterial burdens relative to infections with WT Mtb, this growth attenuation was not observed in infections of *tlr2*^−/−^ mice (Fig. 5). These data provide evidence that TLR2 can act as an important immune modulator during Mtb infection, but this effect is dampened by PPE51 and possibly other bacterial effectors.

Autophagy has increasingly been demonstrated as a vital host pathway for eliminating intracellular bacteria, though its role in controlling Mtb *in vivo* has been questioned. Many canonical autophagy pathway genes do not appear necessary for Mtb control in mice (57, 58). However, this does not consider that Mtb has bacterial effectors inhibiting autophagy during infection and, in that way, renders autophagy ineffective. Along these lines, it has previously been demonstrated that genetic deletion of mycobacterial effectors modulating autophagy can confer a host advantage during Mtb infection (12–15, 23). Our current study demonstrated that autophagy inhibition by Mtb results in enhanced mycobacterial survival in a low-dose murine infection model of TB. Not only do inhibition of TLR2 and autophagy by Mtb reduce the innate immune response to infection, but we also observed inhibition of adaptive immune responses. Infection of mice with Δ*51* demonstrated increased autophagy in the lungs of mice along with enhanced MHC class II antigen presentation and recognition, which is unsurprising given that the known function of autophagy is an efficient cellular process that can degrade pathogens and deliver antigens to MHC class II molecules (59, 60).

Previous studies have also identified PPE51 as an immunogenic target of adaptive immune responses against Mtb (61, 62), which may seem paradoxical given the effects of PPE51 on dampening host responses described in the current study. However, the recognition of PPE51 during infection with Mtb provides further evidence that it must interact with the host immune response and thus is likely to be secreted or at least host exposed. While PPE51 was shown to be secreted by M. smegmatis (12), it has been identified only in cell membrane fractions of Mtb to date. However, one of the core proteins of the ESX-5 secretion system, *eccC5*, demonstrated similar phenotypes of resistance to functionalized carbohydrate derivatives as observed in four *ppe51* mutants of Mtb, providing indirect evidence that PPE51 may be secreted by the ESX-5 type VII secretion system (18).

Since the *ppe51* genetic deletion significantly enhanced CD4^+^ T cell responses to potent Mtb antigens, it will be relevant to test if a *ppe51* deletion in BCG enhances its efficacy as a vaccine. Other successful initial approaches to activate autophagy or TLR2 signaling provide strong evidence that overcoming the bacterial block of these host pathways is a potentially effective therapeutic option (6–8). For example, induction of autophagy with rapamycin demonstrates significant *in vivo* phenotypes in response to mycobacterial infection (63). Indeed, numerous groups have examined rapamycin as a potential therapy for TB, and many host-directed therapies targeting autophagy are being explored as synergistic therapies to current antibiotic treatments against tuberculosis (2–4). Likewise, vaccines enhancing TLR2 responses to mycobacteria are being examined to enhance the protective efficacy of BCG (64). Conversely, the 19-kDa lipoprotein of Mtb, a potent TLR2 ligand, has been shown to inhibit MHC-II expression and antigen presentation in alveolar macrophages in a TLR2-dependent manner (54, 56, 65, 66), suggesting that excessive or prolonged TLR2 signaling may also be detrimental to host immunity. Overall, our findings suggest that Mtb has coevolved effectors to inhibit or subvert the function of TLR2, which in the case of PPE51 may primarily serve to dampen autophagy. Understanding how mycobacterial effectors like PPE51 manipulate host innate immune responses will provide new insights into therapeutic approaches for preventing and treating Mtb.

## MATERIALS AND METHODS

### Bacterial strains and culture conditions.

M. tuberculosis (strain H37Rv) and M. smegmatis (strain mc^2^155) were cultured at 37°C with shaking in Middlebrook 7H9 supplemented with 10% OADC (oleic acid, albumin, dextrose, catalase), 0.5% glycerol, and 0.02% tyloxapol. For growth curves, bacteria were cultured in supplemented 7H9, supplemented 7H9 buffered to pH 5.4 with 150 mM MES (morpholineethanesulfonic acid), or Sauton’s fluid medium (HiMedia) supplemented with 2% glycerol. Middlebrook 7H10 agar medium was supplemented with 10% OADC and 0.5% glycerol with or without antibiotics as per requirements (hygromycin, 50 μg/mL, and kanamycin, 25 μg/mL). Recombinant M. smegmatis strains expressing PPE51 (Msm::51) and the Mtb *ppe51* genetic deletion (Δ*51*) were described previously (12, 30). Briefly, recombinant strains were generated by amplifying full-length *ppe51* from Mtb genomic DNA and cloned in frame into pMV261 (67), an episomal mycobacterial vector with a 3′ His6-HA tag and an *hsp60* promoter. Constructs were electroporated into M. smegmatis or Δ*51* for complementation (Δ*51*C) and selected for kanamycin resistance. The expression of PPE51 was confirmed by immunoblot using an anti-HA antibody. The genetic deletion of *ppe51* in Mtb was conducted by allelic exchange via the previously described specialized phage transduction method (68). Allelic exchange substrates were constructed with approximately 1,000-bp regions upstream and downstream of *ppe51* and directionally cloned into a PacI-containing Escherichia coli cosmid, flanking a hygromycin cassette. The allelic exchange substrate was ligated into the temperature-sensitive mycobacteriophage derived from TM4. Mycobacteriophage-packaged shuttle phasmids were transduced to Mtb H37Rv. Colonies were selected for hygromycin resistance following transduction and screened for gene deletion by PCR.

### Lipid extraction.

Apolar lipid extractions were conducted as previously described (69). Briefly, Mtb was grown to the mid-exponential phase (optical density at 600 nm [OD_600_], 0.4 to 0.8). Apolar lipids were extracted by resuspending bacterial pellets in methanol–0.3% NaCl (10:1) and emulsifying with petroleum ether. The separated top layer (containing apolar lipids) was collected, and emulsions were repeated. Both petroleum ether fractions were combined and dried at 50°C. Apolar lipids were resuspended in chloroform at 5 mg/mL. Lipids were analyzed by TLC using aluminum-backed silica 60 plates (Merck Millipore). Apolar lipids were resolved in petroleum ether-ethyl acetate (98:2 [vol/vol], 3×) and visualized by charring with ceric ammonium molybdate in 6% phosphoric acid.

### Cell culture.

RAW 264.7 macrophages, immortalized BMDM (WT, TLR2^−/−^, TLR4^−/−^, and TLR9^−/−^) (BEI Resources), were maintained in Dulbecco’s modified Eagle medium (DMEM) complete (high-glucose DMEM supplemented with 1% nonessential amino acids, 10% heat-inactivated fetal bovine serum [Corning], and 50 μM β-mercaptoethanol) at 37°C with 5% CO_2_. THP-1 monocytes were maintained in RPMI complete (RPMI containing sodium bicarbonate and l-glutamine supplemented with 1% nonessential amino acids, 1 mM sodium pyruvate, 10 mM HEPES, 50 μM β-mercaptoethanol, and 10% heat-inactivated fetal bovine serum) at 37°C with 5% CO_2_. Monocytes were differentiated to macrophages using 10 μg/mL phorbol 12-myristate 13-acetate (PMA) in RPMI complete for 48 h. The attached cells were then washed in RPMI complete and rested for a minimum of 4 h and up to overnight before infection.

BMDM were prepared as previously described from 6-week-old C57BL/6J or C57BL/6J TLR2^−/−^ mice (70). Briefly, marrow was flushed from the tibia and femur and collected aseptically. Cells were cultured in non-tissue-culture-treated 100- by 20-mm petri dishes at a density of 2 × 10^6^ cells/dish in BMDM medium (RPMI complete containing 100 U/mL penicillin, 100 μg/mL streptomycin, and 15% L929 fibroblast-conditioned medium) for differentiation for 6 days. On day 6, adherent cells were collected by detachment using phosphate-buffered saline (PBS) at 4°C for 30 min and seeded in 12-well plates in RPMI complete.

All macrophage assays were conducted in 12-well plates, seeded at 5 × 10^5^ cells/well (RAW 264.7) or 1 × 10^6^ cells/well (THP-1 and BMDM). Msm and Mtb strains were grown to an OD_600_ of 0.6 to 0.8 and infected at an MOI of 10 unless otherwise stated. Inocula were prepared by centrifugation at 800 × *g* for 8 min to remove large clumps. Infection was carried out for 3 h in DMEM complete at 37°C with 5% CO_2_ for 4 h for THP-1 macrophages and primary cells in RPMI complete. Cells were then washed three times with PBS, followed by treatment with 50 μg/mL gentamicin in DMEM or RPMI complete for 1 h to kill extracellular bacteria. Macrophages were then washed three times with PBS and incubated for indicated times in DMEM or RPMI complete containing 20 μg/mL gentamicin. Cells were harvested at indicated time points in radioimmunoprecipitation assay (RIPA) buffer (150 mM NaCl, 1% NP-40 or Triton X-100, 0.5% sodium deoxycholate, 0.1% SDS, 50 mM Tris-HCl [pH 8.0], 20 mM Tris-HCl [pH 7.5]) for plating for intracellular survival or analysis of autophagy induction by immunoblotting. For CFU enumeration, lysates were serially diluted and plated on 7H10 agar. To determine bacterial survival, macrophages were lysed 4 h postinfection (after an initial three washes with PBS) and plated for enumeration. The number of phagocytosed bacteria at this time was used to normalize numbers of bacteria across strains and calculate fold change.

### Immunoblotting.

Cellular protein was prepared in 1× RIPA buffer, and its concentration was determined by bicinchoninic acid (BCA) assay (Pierce). One to 10 μg of protein was resolved on 12% SDS-PAGE gels at 180 V for 30 to 40 min. Proteins were transferred to 0.2 μm polyvinylidene difluoride (PVDF) membranes using a Bio-Rad Transblot Turbo at 2.5 A and 25 V for 5 to 10 min, depending on molecular weight. PVDF membranes were blocked in 5% nonfat dry milk in 1× Tris-buffered saline plus 0.2% Tween 20 (TBST) or OneBlock Western-CL blocking buffer (Genesee Scientific) for LC3B blots at 4°C overnight. Primary antibodies at a 1:5,000 dilution were incubated for 2 h at room temperature in TBST. Anti-rabbit IgG-horseradish peroxidase (HRP) antibody (Cell Signaling Technology) (1:10,000) was added to membranes for 45 min in TBST. Proteins of interest were revealed using Clarity ECL (Bio-Rad) according to the manufacturer's instructions. Films were scanned, and densitometric analysis was conducted with ImageJ software (https://imagej.nih.gov/ij). The protein of interest was normalized to β-actin, GAPDH (glyceraldehyde-3-phosphate dehydrogenase), or β-tubulin loading controls to calculate levels of autophagy (71).

### Antibodies and other reagents.

Antibodies were purchased from Cell Signaling Technology unless indicated otherwise and are listed with details in Table S1. Anti-HA peroxidase-conjugated antibody (final concentration, 25 mU/mL) was purchased from Sigma-Aldrich (clone 3F10). All other reagents and media were purchased from Sigma-Aldrich unless otherwise stated.

10.1128/mBio.02974-21.1 TABLE S1 Antibodies used, with details. Download Table S1, DOCX file, 0.01 MB. Copyright © 2022 Strong et al. 2022 Strong et al. https://creativecommons.org/licenses/by/4.0/ This content is distributed under the terms of the Creative Commons Attribution 4.0 International license.

### ROS accumulation determination.

ROS were detected in macrophages using the CellROX reagent (Thermo Fisher Scientific) as per the manufacturer’s instructions. Briefly, CellROX reagent was added to macrophage monolayers 15 to 30 min before collection. RAW 264.7 macrophages were collected by lifting with trypsin, while BMDM were collected using ice-cold PBS. Collected cells were washed once in ice-cold PBS and finally resuspended in 4% paraformaldehyde (PFA) for 48 h. Samples were acquired on an Accuri C6 Plus flow cytometer (BD), and data were analyzed using FlowJo.

### Generation of inducible PPE51-expressing macrophages.

RAW 264.7 macrophages were stably transduced with the second-generation lentiviral Tet-on vector pInducer20 (72) in which PPE51 from Mtb had been cloned using gateway cloning. RAW 264.7 macrophages were transduced using recombinant lentivirus. Lentivirus was generated using ViromerRed (OriGene Technologies, Maryland) according to the manufacturer's instructions in the human embryonic kidney (HEK) 293T cells. Briefly, expression constructs (pInducer, 1.64 pmol), packaging (psPAX2, 1.3 pmol), and envelope (pMD2.G, 0.72 pmol) plasmids were added to ViromerRed and then added to cells. HEK 293T cell culture supernatant was collected at 48 h posttransfection, and Polybrene was added at 10 μg/mL to generate suspensions for transduction. The recombinant lentivirus suspensions were added to RAW 264.7 macrophages and incubated for 24 h, after which medium containing lentivirus was removed, and fresh complete Dulbecco's Minimal Essential Medium (DMEMc) was added. At 48 h after the addition of lentivirus, macrophages were treated with 400 μg/mL G418. Single G418-resistant cells were expanded and assayed for PPE51 expression after induction with 500 ng/mL aTCN for 16 h.

### Atg16L1 knockdown with shRNAs.

Atg16L1 short hairpin RNA (shRNA) constructs were purchased from Horizon Inspired Cell Solution, constructed by the RNAi Consortium (TRC-Mm1.0; clone IDs TRCN0000173438, TRCN0000175121, TRCN0000175371, TRCN0000175562, and TRCN0000176385). The lentiviral shRNA plasmids were transfected into HEK293T cells to produce lentivirus packed with shRNA.

Single puromycin-resistant cells were selected with 10 μg/mL of puromycin after lentivirus infection and then subjected to standard expansion. Knockdowns were functionally validated by Western blotting.

### Annexin-V and 7-AAD staining.

THP-1 monocyte-derived macrophages were seeded and infected as described above. At 72 h postinfection, cells were washed with PBS and collected using trypsin. Cells were stained using the GFP-Certified apoptosis/necrosis detection kit (Enzo Life Sciences, USA) as per the manufacturer’s directions. Briefly, once collected, cells were washed once in ice-cold PBS, resuspended in dual detection reagent (annexin-V and 7-aminoactinomycin D [7-AAD]), and incubated for 15 min. Cells were washed once more and resuspended in 4% PFA for 48 h. Samples were acquired on an Accuri C6 Plus flow cytometer (BD), and data were analyzed using FlowJo.

### ELISA.

At indicated time points, cell culture supernatants were collected for cytokine analysis. BioLegend enzyme-linked immunosorbent assay (ELISA) Max Deluxe kits were used as per the manufacturer's instructions. Briefly, plates were coated in capture antibody for 16 h and then incubated with blocking buffer for 1 h at room temperature. A total of 100 μL of standards or culture supernatant was added and incubated for 2 h at room temperature. The detection antibody was incubated for 1 h, followed by avidin-HRP for 30 min. TMB (3,3′,5,5′-tetramethylbenzidine) substrate was incubated for 20 min, and sulfuric acid was added to stop the reaction. Absorbance was read at 450 nm, and background absorbance (570 nm) was subtracted.

### Confocal microscopy.

RAW264.7 LC3-GFP cells were infected with H37Rv-DsRed (Mtb::DsRed) or Δ*51*-DsRed (Δ*51*::DsRed) at an MOI of 10. Cells were washed and fixed with 4% PFA at 24 h postinfection. ProLong Gold antifade with DAPI (4′,6-diamidino-2-phenylindole; Cell Signaling Technologies) was used as the mounting medium. The confocal images were acquired by using an A1 Nikon confocal microscope (Nikon, Japan). Pearson's correlation was used to quantify colocalization and was analyzed by using NIS Elements software (Nikon, Japan).

### *In vivo* infection.

All animal studies were approved by the institutional animal care and use committee of the University of Texas Medical Branch. Female C57BL/6J and TLR2^−/−^ (no. 022507) mice were obtained from The Jackson Laboratory between 6 and 8 weeks of age. Mice were infected with ∼200 CFU Mtb intranasally. For intranasal infection, mice were anesthetized with isoflurane, and 12.5 μL of Mtb inoculum was delivered to each naris by micropipette. The Mtb inoculum was prepared from a freshly grown culture, which was washed twice, centrifuged at 800 × *g*, and diluted to an optimal density. Infection dose was confirmed 1 day postinfection, and bacterial burdens in lungs and spleens of mice were determined 2 and 4 weeks postinfection. For CFU enumeration, lungs and spleens of individual mice were aseptically collected into 2 mL PBS and homogenized using a probe homogenizer. Lysates were then serially diluted and plated on 7H10.

### T cell response.

Splenocytes were isolated from C57BL/6J mice infected with Mtb. Single cells were obtained and suspended in fluorescence-activated cell sorting (FACS) buffer (2% bovine serum albumin [BSA] in PBS). Cells were surface stained for CD4 and CD8, and Fc receptors were blocked with CD16/32 (a list of antibodies is provided in Table S1) in FACS buffer for 30 min on ice. Cells were washed and stained with peptide-loaded MHC class II tetramers in FACS buffer for 30 min at 37°C. Samples were subsequently washed twice with PBS and fixed in 4% PFA. Quantification of IFN-γ- or TNF-α-producing CD4^+^ cells in response to antigen stimulation was done by intracellular cytokine staining. Briefly, 1 × 10^6^ splenocytes/mL were stimulated with synthetic peptides (GenScript) or whole-cell lysate (BEI) at 10 μg/mL for 11 h. Cytokine secretion was inhibited by the addition of GolgiStop for a further 5 h. Cells were then Fc blocked and stained for viability, CD4, and CD8 in FACS buffer for 20 min at 4°C. Cells were washed and fixed and permeabilized as per the manufacturer’s instructions (BD). Fluorochrome-conjugated antibodies against IFN-γ and TNF-α were added to cells in permeabilization buffer and incubated for 1 h at 4°C. Cells were washed and fixed in 4% PFA. All samples were acquired on a Fortessa flow cytometer (BD) and analyzed using FlowJo software.

### Statistical analysis.

GraphPad Prism 8 was used for all analyses. Analysis of variance (ANOVA) was used to determine significance, with Dunnett’s correction for multiple comparisons. A *P* value of <0.05 was considered significant.
