# Supplementary figures and images for: Mycobacterium tuberculosis PPE51 Inhibits Autophagy by Suppressing Toll-Like Receptor 2-Dependent Signaling
Source: mBio. 2022 Apr 25;13(3):e02974-21. doi: 10.1128/mbio.02974-21 (PMC9239179; doi:10.1128/mbio.02974-21)

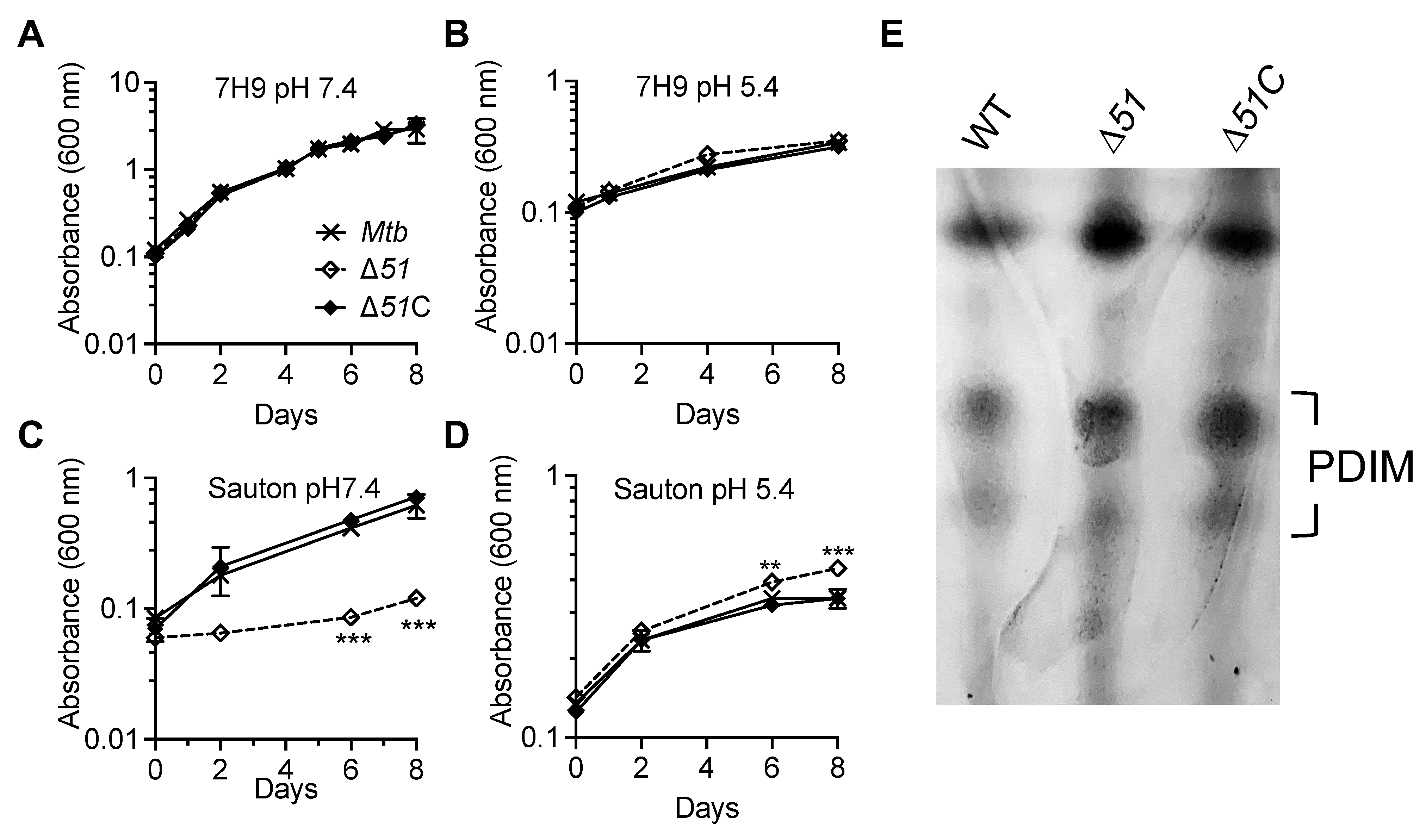

Supplement: FIG S1 [file mbio.02974-21-sf001.tif]

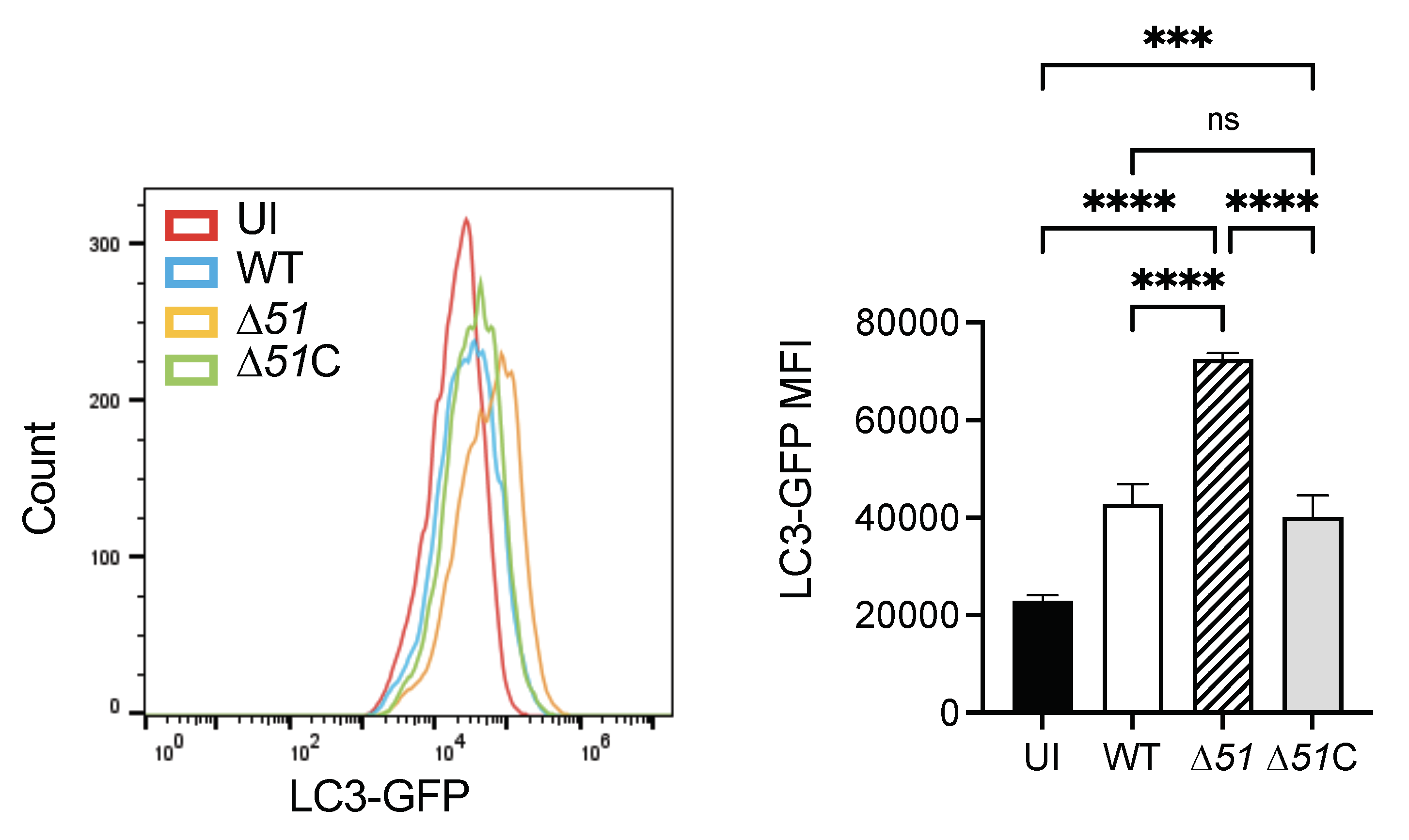

Supplement: FIG S2 [file mbio.02974-21-sf002.tif]

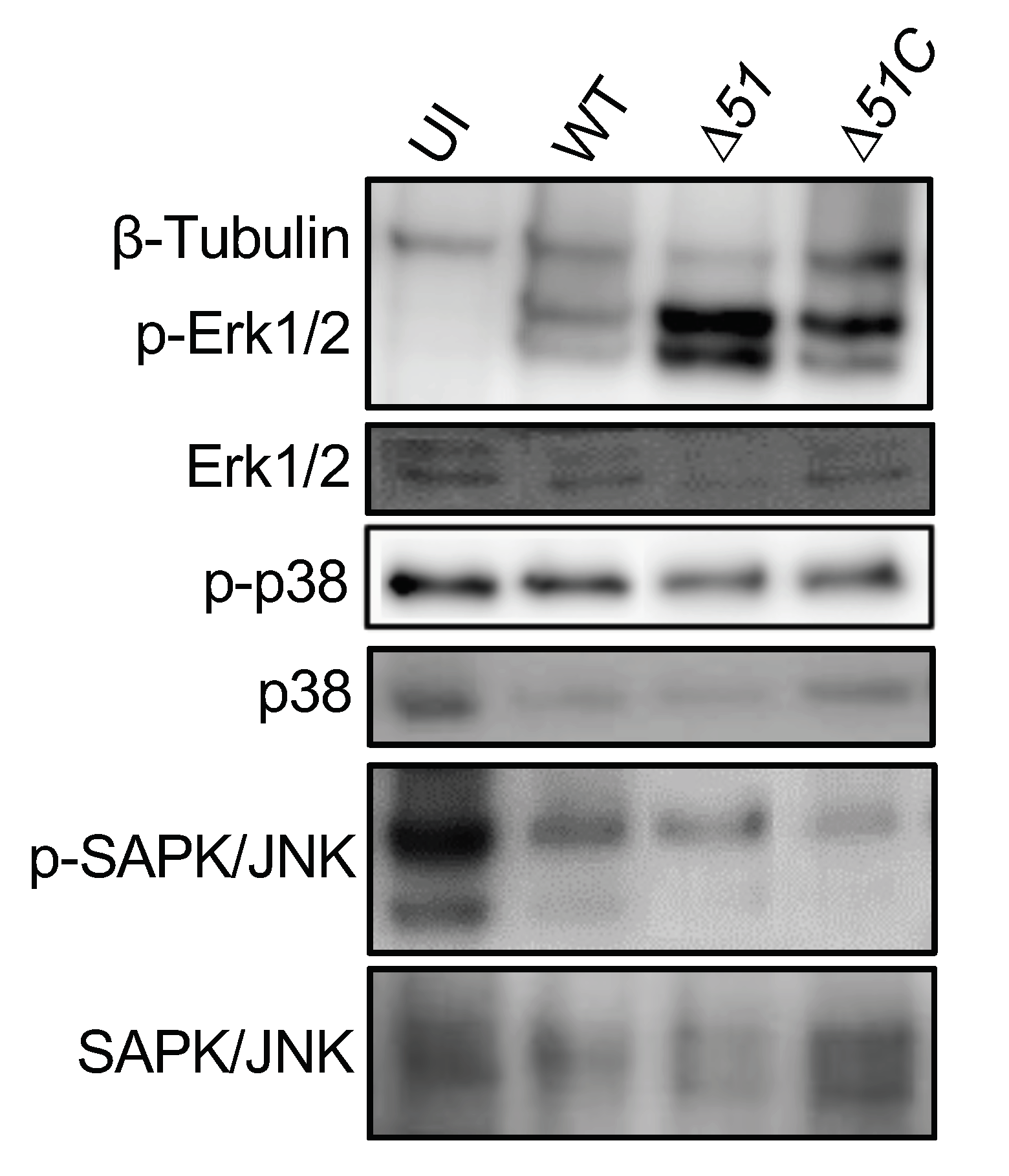

Supplement: FIG S3 [file mbio.02974-21-sf003.tif]

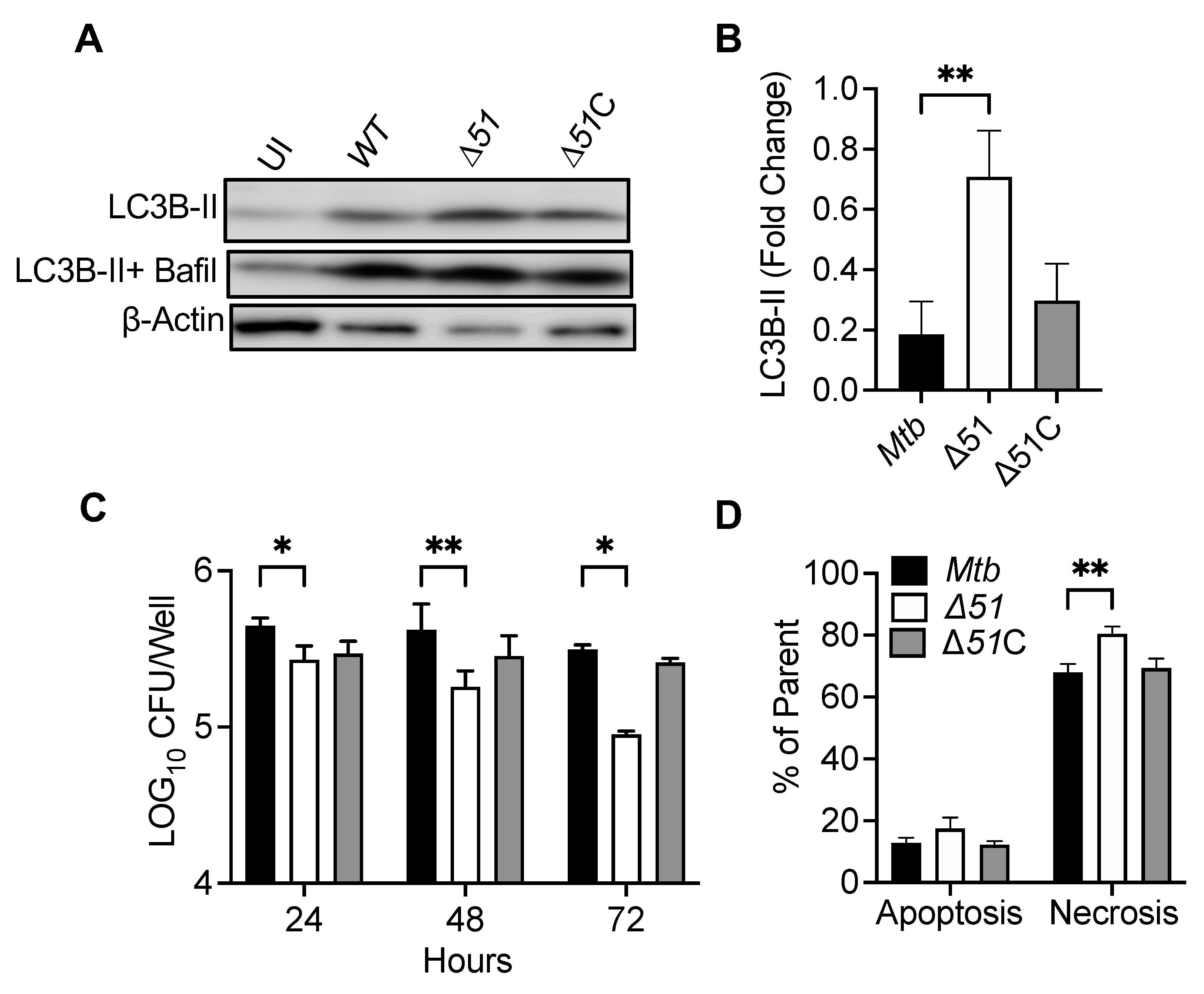

Supplement: FIG S4 [file mbio.02974-21-sf004.tif]

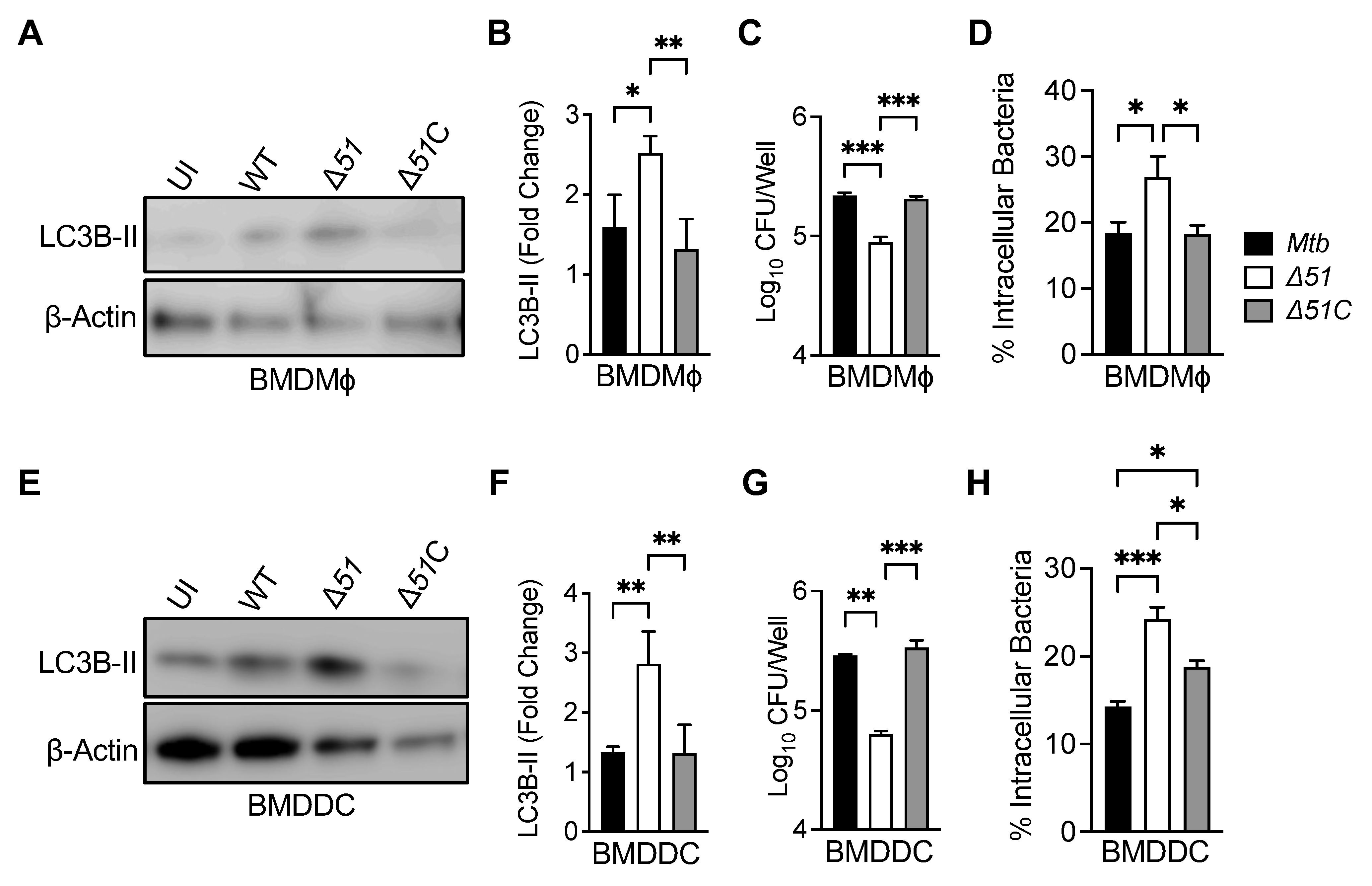

Supplement: FIG S5 [file mbio.02974-21-sf005.tif]

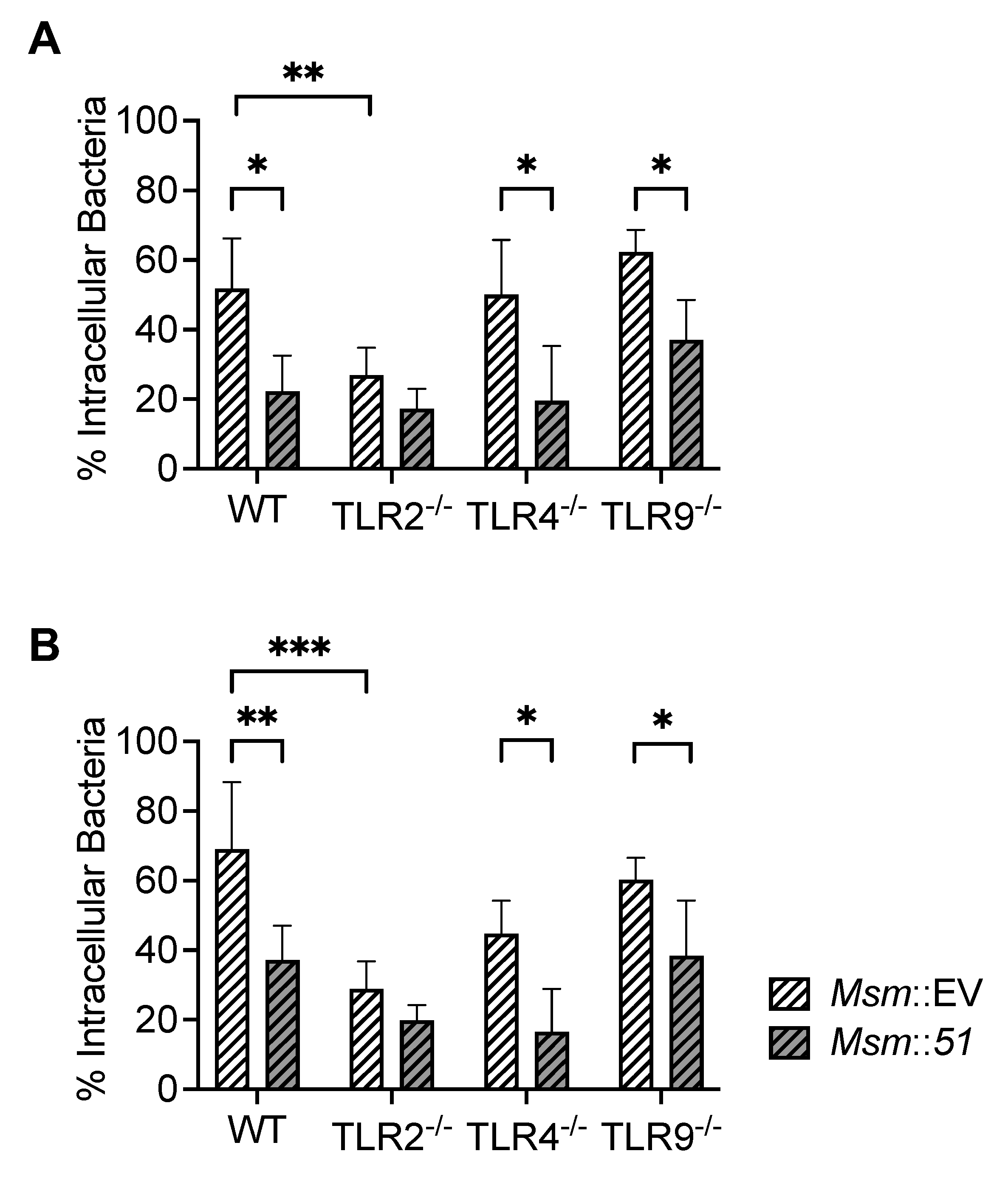

Supplement: FIG S6 [file mbio.02974-21-sf006.tif]

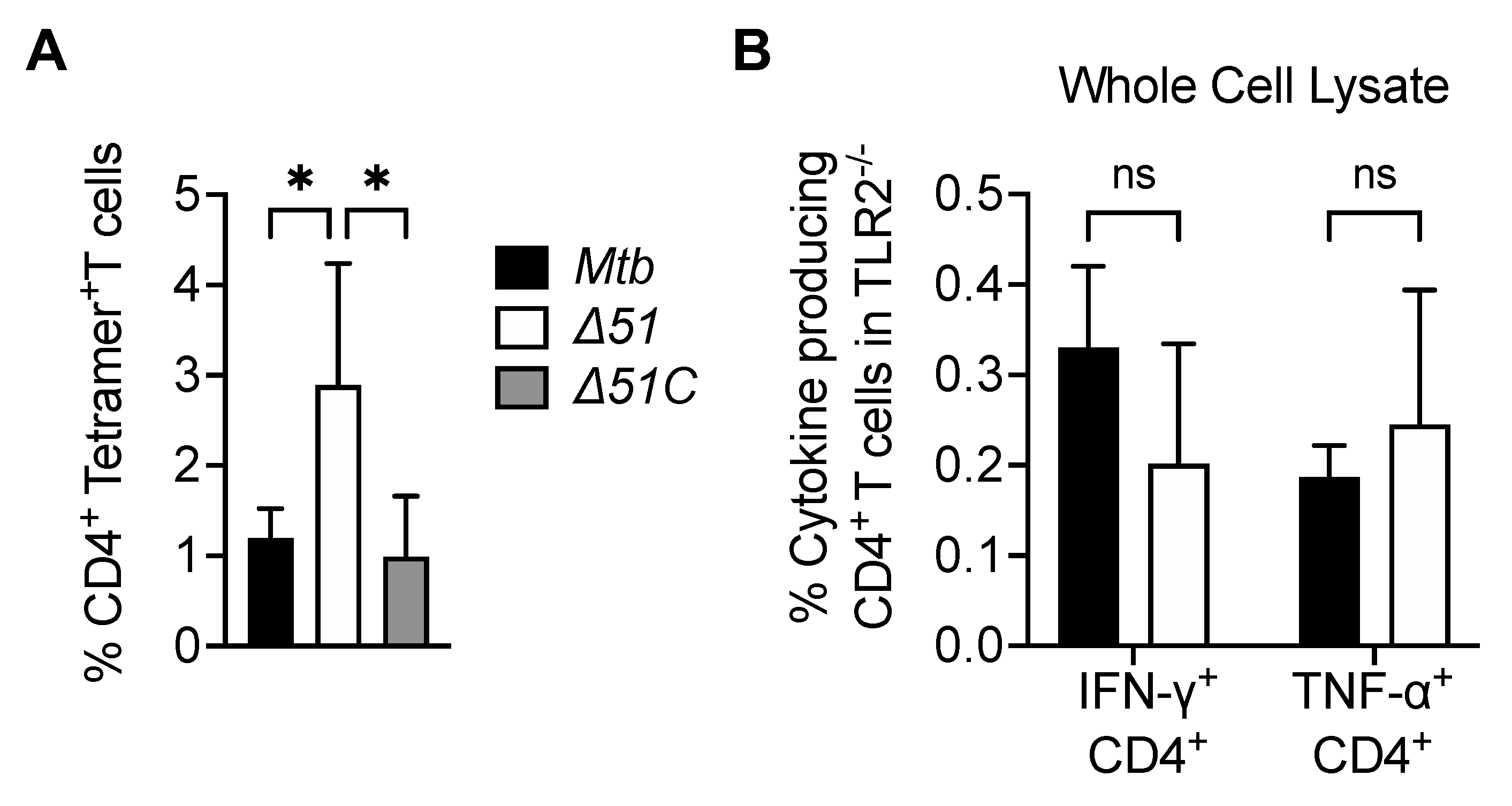

Supplement: FIG S7 [file mbio.02974-21-sf007.tif]
