# Supplementary material for: Mycobacterium tuberculosis PPE51 Inhibits Autophagy by Suppressing Toll-Like Receptor 2-Dependent Signaling
Source: mBio. 2022 Apr 25;13(3):e02974-21. doi: 10.1128/mbio.02974-21 (PMC9239179; doi:10.1128/mbio.02974-21)
Supplement: TABLE S1 [file mbio.02974-21-st001.docx]

**Supplementary Table 1: List of Antibodies**

| **Antibody** | **Company** | **Catalog Number** |
| --- | --- | --- |
| LC3B (D11) | Cell Signalling Technology | 3868 |
| β-Actin | Cell Signalling Technology | 4970 |
| HA-Peroxidase | Sigma | 12013819001 |
| Rabbit IgG-HRP | Cell Signalling Technology | 7074 |
| p-ERK1/2 | Cell Signalling Technology | 4370 |
| β-Tubulin | Cell Signalling Technology | 2128 |
| GAPDH | Cell Signalling Technology | 5174 |
| p62/SQSTM1 | Cell Signalling Technology | 5114 |
| Fc Block | Biolegend | 101302 |
| CD4 - Pacific Blue | Biolegend | 100427 |
| CD8 - AlexaFluor 488 | Biolegend | 100726 |
| IFN-γ - APC | Biolegend | 505809 |
| TNF-α - PE | Biolegend | 506305 |
